# Genetic dissection of morphological variation in rosette leaves and leafy heads in cabbage (*Brassica oleracea* var. *capitata*)

**DOI:** 10.1007/s00122-022-04205-w

**Published:** 2022-09-03

**Authors:** Jorge Alemán-Báez, Jian Qin, Chengcheng Cai, Chunmei Zou, Johan Bucher, Maria-João Paulo, Roeland E. Voorrips, Guusje Bonnema

**Affiliations:** 1 Plant Breeding, Wageningen University and Research, Droevendaalsesteeg 1, Wageningen, The Netherlands; 2 Centre for Crop Systems Analysis, Wageningen University and Research, PO Box 430, 6700 AK Wageningen, The Netherlands; 3 Biometris, Wageningen University and Research, Droevendaalsesteeg 1, Wageningen, The Netherlands

## Abstract

**Key message:**

Correlations between morphological traits of cabbage rosette leaves and heads were found. Genome-wide association studies of these traits identified 50 robust quantitative trait loci in multiple years. Half of these loci affect both organs.

**Abstract:**

Cabbage (*Brassica oleracea* var. *capitata*) is an economically important vegetable crop cultivated worldwide. Cabbage plants go through four vegetative stages: seedling, rosette, folding and heading. Rosette leaves are the largest leaves of cabbage plants and provide most of the energy needed to produce the leafy head. To understand the relationship and the genetic basis of leaf development and leafy head formation, 308 cabbage accessions were scored for rosette leaf and head traits in three-year field trials. Significant correlations were found between morphological traits of rosette leaves and heads, namely leaf area with the head area, height and width, and leaf width with the head area and head height, when heads were harvested at a fixed number of days after sowing. Fifty robust quantitative trait loci (QTLs) for rosette leaf and head traits distributed over all nine chromosomes were identified with genome-wide association studies. All these 50 loci were identified in multiple years and generally affect multiple traits. Twenty-five of the QTL were associated with both rosette leaf and leafy head traits. We discuss thirteen candidate genes identified in these QTL that are expressed in heading leaves, with an annotation related to auxin and other phytohormones, leaf development, and leaf polarity that likely play a role in leafy head development or rosette leaf expansion.

**Supplementary Information:**

The online version contains supplementary material available at 10.1007/s00122-022-04205-w.

## Introduction

Cabbage (*Brassica oleracea* ssp. *capitata*) is an important leafy vegetable and a healthy source of mineral nutrients, crude fibre, and vitamins, consumed worldwide (Lv et al. 2014). The global production of cabbage and other *Brassicas* was more than 70 million tons in 2020 (fao.org/faostat). The economically important part of the cabbage plant is the leafy head, formed by densely packed leaves. Different cabbage crop types are mainly categorized by the differences in head shape, surface, and colour. White cabbages have smooth leaves that form tight round or flat leafy heads; pointed cabbages also have smooth leaves but have a cone-like head; savoy cabbages have rugose leaves and looser heads; red cabbages have compact heads with often oblong shapes and are distinguished by their colour. Moreover, distinct morphological and agronomic traits are selected in cabbages for different uses (fresh, fodder, industry, or storage), quality traits (colour, taste, crispiness), time until head maturity (early, middle, and late), and the growing season (summer, autumn, and winter).

The leafy head is a domesticated trait that provides a compact and therefore transportable and storable form of leaves. The heading trait occurs also in Chinese cabbage (*Brassica rapa* ssp. *pekinensis*), lettuce (*Lactuca sativa* ssp. *capitata*), radicchio (*Cichorium intibus*), and other crops. To produce the leafy head, cabbage plants go through four vegetative stages: seedling development, rosette formation, leaf folding, and head formation. At the seedling stage, the first true leaves with a roundish shape and long petioles appear. At the rosette stage, the cabbage plant produces flat and large leaves, while petioles disappear, that serve as the major photosynthetic organs and become supporters of the growing leafy head. At the folding stage, the first inward curved leaves, with also an important role in photosynthesis, appear and each of the subsequent leaves shows an increased curvature. At the heading stage, the emerging leaves show an extreme inward curvature causing the overlapping between leaves around the shoot apex. The outer heading leaves constrain the inner heading leaves forcing them to fill the leafy head.

Leaf development is extensively studied in the model plant *Arabidopsis thaliana* and several scientific reviews are available (Du et al. 2018; Yang et al. 2018; Nikolov et al. 2019). Additionally, Karamat et al. 2021 also reviewed the genetic factors involved in leaf growth in Chinese cabbage. Leaves develop from the peripheral zone in the shoot apical meristem (SAM). In this zone, auxin maxima among the cell population triggers the production of the leaf primordia. Auxin gradients are formed by importer AUXIN RESISTANT (AUX1) and efflux carriers PIN-FORMED1 (PIN1) (Bayer et al. 2009; Kalve et al., 2014). After leaf primordia initiation, the adaxial–abaxial, the proximal–distal, and the medial–lateral polarities are established. The ad/abaxial polarity is determined by domain-specific transcription factors families. The adaxial domain is mainly determined and maintained by a group of plant-specific homeodomain/leucine zipper (HD-ZIP) transcription factors, whereas the abaxial domain by the KANADI (KAN) and YABBY (YAB) families together with the AUXIN RESPONSE FACTORS ARF3 and ARF4. Adaxial and abaxial genes and other genetic factors such as small RNAs like miR165/166 and transacting small interfering RNA (ta-siRNA) interact at both protein and transcript levels to create leaf ad/abaxial polarity (Chitwood et al. 2007, 2009; Nogueira et al. 2007). Differences in cell growth rates between the ad/abaxial sides, and between the leaf centre and margins induce leaf curvature (Nath et al. 2003 and Mao et al. 2014). Leaf curvature is nicely studied in the model plant Arabidopsis (Liu et al. 2011; Yamaguchi et al. 2012; Sandalio et al. 2016) and Chinese cabbage (Li et al. 2019). Little is known about the leafy head trait in cabbage (*B. oleracea*). Most studies about the heading trait in *Brassicas* have been conducted in Chinese cabbage (*B. rapa*). In cabbage (Lv et al. 2014) and Chinese cabbage (Yu et al. 2013; Mao et al. 2014; Sun et al. 2018), many QTLs associated with head traits have been identified. This was done by using a double haploid population derived from two heading cabbages with different maturity times and geographical origins (Lv et al. 2014) or using both F2 populations (Sun et al. 2018) and recombinant inbred line (RIL) populations (Yu et al. 2013; Mao et al. 2014) derived from multiple crosses between heading and non-heading morphotypes. These studies indicates that the leafy head is a quantitative trait controlled by multiple genes. In 2016, Cheng et al. compared genomic variations between heading and non-heading morphotypes to identify selective sweeps in both *B. rapa* and *B. oleracea* involved in the leafy head trait. Interestingly, several candidate genes with roles in leaf ad/abaxial polarity determination were identified. These included ARF3.1 and 4.1 (*BrARF3.1* and *BrARF4.1*), KANADIs (*BrKAN2.1*, *BrKAN2.3,* and *BoKAN2.2)*, and a member of the HD-ZIP III gene family (*BoATHB15.2*). Liang et al. (2016) provided additional genetic evidence for roles of these genes in the head formation in Chinese cabbage. Additionally, Liang et al. (2016) propose that the RNA-DEPENDENT RNA POLYMERASE 6 (*BrRDR6)* involved in the production of tasiRNAs (Moon and Hake 2011) and HYPONASTIC LEAVES1 (*BrHYL1.1*) that facilitates the maturation of micro-RNAs (Kurihara et al. 2006) also participate in the head formation. In yet another study, Mao et al. (2014) found that rosette leaf shape and leafy head shape in Chinese cabbage were correlated and that a mutation of the *TCP4* gene in the miR319a recognition site affected both cell division and cell proliferation, resulting in different rosette leaf and head shapes.

Since 2014, more and more genomic resources became available for *B. oleracea* studies. In 2014, the first reference genome “JZS v1” (Liu et al. 2014) was published, followed by an improved version “JZS v2” (Cai et al. 2020) with a 20% increase in the total length of the sequence assembly. In addition, Cai et al. (2022) and Mabry et al. (2021) studied the genetic variation in large *B. oleracea* collection with genomic data. Cai et al*.* generated 330,383 high-quality single nucleotide polymorphism (SNP) markers. We filtered these markers to select only the SNPs occurring among the subset of heading cabbage accessions. These filtered SNPs were used as input for Genome-Wide Association Studies (GWAS) to identify the genetic elements participating in a trait of interest. This study aims to assess the genetic diversity and morphological variation for both rosette leaf and head traits in more than three hundred cabbage accessions representing both modern F1 hybrid cultivars and open-pollinated gene bank accessions, different crop types (white, pointed-headed, savoy, and red cabbage), and diverse geographic origins. This information is combined and used to reveal the correlations between the traits and for a GWAS to identify marker associations with rosette leaf and head traits. For a subset of the associations, candidate genes involved in cabbage leaf development and head formation are listed. For this, we hypothesize that genes involved in leaf polarity, leaf growth (cell division and expansion), and leaf development that are expressed in leaves in the heading stage might regulate the leafy head trait in cabbage.

## Material and methods

### Plant material

In this project, a collection of 308 cabbage accessions from diverse geographical origins and genetic backgrounds, obtained from germplasm banks and breeding companies (mostly F1 hybrids) was studied (Supplementary Table S1 and Cai et al. 2022). This cabbage collection included white, red, savoy, and pointed accessions; early, middle, and late types; for industry, fresh, and storage usage. Three field trials were conducted during the 2017, 2018, and 2019 spring–summer seasons for the phenotyping of cabbage rosette leaves and head traits. Table 1 lists the characteristics of these three field trials. Each trial included a different subset of the cabbage collection; the 2017 trial included 291 cabbage accessions, 2018 181, and 2019 247 (Supplementary Table S1); 139 cabbage accessions were included in all three years (Supplementary Table S1). These 139 accessions are referred to as “common accessions”. Cabbage seeds were sown in germination trays in sandy soil in a greenhouse, during the 2nd week of April for the 2017 trial, and during the 2nd week of May for 2018 and 2019. Four weeks after sowing, the cabbage seedlings for each trial were transplanted to the open field (river clay soil) at Wageningse Afweg (51.953 N, 5.638 E), Netherlands. In each year a different part of the field was used. All field trials followed a complete randomized block design with two blocks (A and B). Within each block, each cabbage accession was represented by one plot of five plants. Each plot served as a biological replicate. The position of each accession plot within the field was labelled in a *X* and *Y* coordinate system. The individual plants in the same plot shared the same coordinates. Table 1 and supplementary Tables S2, S3, and S4 list the weather conditions of each year, extracted from the Royal Dutch Meteorological Institute (KNMI) (https://www.knmi.nl/, accessed 15 November 2021), of the Geelen station (52.056 N, 5.873 E) located at 21.75 km away from the trial field.

**Table 1:** Description and weather conditions (in average) of the three field trials

Field trial year	2017	2018	2019
Number of cabbage accessions	291	181	247
Common cabbage accessions	139		
Sowing date	11/Apr/17	9/May/18	6/May/19
Transplanting to open field date	8/May/17	4/Jun/18	3/Jun/19
Field location	Wageningse Afweg (51.953 N, 5.638 E), Netherlands		
Blocks per trial	Two (A and B)		
Plots per block	One plot for each accession		
Plants per plot	Five		
Daily mean temperature (°C)	14.9	16.9	15.4
Daily mean minimal temperature (°C)	9.2	10.4	9.4
Daily mean maximal temperature (°C)	20.2	22.9	21.1
Global radiation (in J/cm^2^)	1500.39	1753.6	1692
Daily precipitation amount (in 0.1 mm)	22.7	15.7	19.1

### DNA isolation, sequencing, and SNP calling

In this study, we utilized the genotypic dataset produced by Cai et al. 2022. DNA isolation, sequence-based genotyping, and variant calling were as described by Cai et al. 2022. Cai and colleagues genotyped 912 globally distributed accessions representing ten *B. oleracea* morphotypes, wild *B. oleracea*, and wild C9 *Brassica* species. The 308 cabbage accessions utilized in this study are a subset of this large collection. The amount of resequencing data generated from this cabbage subset is, on average, 494 Mb per accession (4.12 million single-end reads) (Supplementary Table S1). Basically, this resequencing data (Illumina reads) were aligned to the JZS v1 cabbage reference genome (Liu et al. 2014), which is composed of 385 Mb of sequences that were anchored to the nine physical chromosomes of cabbage and 131 Mb unanchored scaffolds that were grouped into a so-called chromosome zero (C00). In total, Cai et al. 2022 detected 742,169 raw biallelic single nucleotide polymorphisms (SNPs) using SAMtools v1.9 (Li et al. 2009) and BCFtools v1.10 (Li 2011). To obtain high-quality SNPs, Cai et al. 2022 filtered these raw SNPs with strict criteria. If a SNP was supported by less than 3 reads in an accession it was scored as a missing value. If two alleles were supported by at least two reads the SNP was scored as heterozygous. Otherwise, the SNP was scores as homozygous. The filtering process resulted in 330,383 high-quality SNPs. The SNP allele codes were 0: homozygous for the reference allele, 1: heterozygous, and 2: homozygous for the alternative allele. In our study, three independent sets of markers were created from the high-quality SNPs generated by Cai et al. 2022, one for each of the three cabbage data sets (2017, 2018, and 2019). As each set of SNPs was filtered for a genotyping rate ≥ 80% and a minor allele frequency (MAF) ≥ 2.5%, this resulted in slightly different SNP sets for the different years. This SNP filtering process was performed using the BCFtools v1.10 software (Li 2011) with parameter settings of Cai et al. 2022.

### Genetic diversity assessment

The genetic variation for each of the three cabbage subsets was assessed using DARwin v6.0.21 software (Perrier et al*.* 2006). For each year’s SNP dataset, a PCO (Principal Coordinate) analysis, based on euclidean dissimilarities, was performed with default parameters. Each PCO analysis produced a dissimilarity matrix.

### Phenotyping of rosette leaves and head traits

The phenotyping of cabbage plants occurred at two time points: late rosette stage from 60 to 75 days after sowing (DAS) and heading stage from 80 DAS onwards. Rosette leaves and heads were phenotyped at the heading stage for all three trials (Table 2). Additionally, rosette leaves were phenotyped at the rosette stage for 2018 and 2019 (Table 2). To phenotype rosette leaves, the largest rosette leaf available was detached from each of three most similar plants (out of five) per plot. Similarly, the cabbage heads were also harvested from the three most similar plants per plot. The moment of harvesting rosette leaves and heads was determined differently in each trial (Table 2). The trial of 2017 had only one phenotyping moment, at heading stage (91–127 DAS). In this trial, rosette leaves and heads were harvested simultaneously per accession from both blocks (A and B) when the plants of the accession had reached head maturity. Head maturity was assessed by the tightness and compactness of the leafy head. The trial of 2018 had three phenotyping moments. The rosette leaves were harvested at rosette stage (62–70 DAS) and heading stage (83–86 DAS); the heads were harvested at heading stage (111–124 DAS). This meant that in contrast to 2017, in 2018 head maturity of individual accessions was not used to determine the time of harvest. In these three harvests, block A (all four crop types) was phenotyped first, followed by block B. The trial of 2019 had also three phenotyping moments. The accessions were selected differently for the phenotyping of rosette leaves and heads. The rosette leaves were phenotyped in rosette stage (70–74 DAS) and at heading stage (84–88 DAS) like in 2018, but each crop type was completely phenotyped in both block A and block B before moving to the next crop type. The heads were harvested in heading stage (91–112 DAS early types; 134 DAS late types), the heads were again harvested simultaneously per accession from both blocks when the plants of the accession had reached head maturity like in 2017. A different set of traits was phenotyped in each of the three years (Table 2); the traits scored in all three years are defined as “common traits”. In all trials, cabbage plants that showed “cracking” of the outer leaves due to pressure from the younger leaves inside the head were excluded for the head traits phenotyping and analysis. Thus, 249 accessions were utilized for 2017, 163 for 2018, and 218 for 2019.

**Table 2:** Traits scored and scoring dates in days after sowing (DAS)

			2017			2018			2019		
			Scored	Harvest (DAS)	Description	Scored	Harvest (DAS)	Description	Scored	Harvest (DAS)	Description
Leaf traits	Rosette stage	Leaf area (cm^2^)	×	×	×	✓	**62** *S, R, P and W* (BA); **63** *W* (BA); **70 ***S, R, P and W* (BB)	Leaves and heads were harvested in a same time window	✓	**70** *R* (BA/BB); **71** *P* (BA/BB) and *W* (BA); **72** *W* (BA/BB); **73** *W* (BB); **74** *S* (BA/BB)	Leaves were harvested in a same time window
		Leaf length (cm)	×			✓			✓		
		Leaf width (cm)	×			✓			✓		
		Leaf ratio (length/width)	×			✓			✓		A crop type was completely phenotyped in BA and then in BB before moving to the next crop type
	Heading stage	Leaf area (cm^2^)	✓	**91, 93, 94, 97, 106, 107 and 127**	Leaves and heads were phenotyped simultaneously	✓	**83** *S, R, P and W* (BA); **84** *W* (BA); **86** *S, R, P and W* (BB)		✓	**84** *W* (BA); **85** *W* (BA/BB); **86** *W* (BB) and *P* (BA/BB); **87** *R* (BA/BB); **88** *S* (BA/BB)	
		Leaf length (cm)	✓			✓			✓		
		Leaf width (cm)	✓			✓			✓		
		Leaf ratio (length/width)	✓			✓		BA was fully phenotyped before BB	✓		
		Rosette leaf number	×		Accessions were selected based on head maturity	×			✓		
Head traits	Heading stage	Head area (cm^2^)	✓			✓	**111** *S and R* (BA), **112** *R* (BA), **117** *W* (BA); **118** *S and R* (BB), **119** *R* (BB), **124** *W* (BB)		✓	**91–112 and 134**	Accessions were selected for head harvesting based on maturity
		Head height (cm)	✓			✓			✓		
		Head width (cm)	✓		BA and BB were phenotyped simultaneously	✓			✓		
		Head ratio (height/width)	✓			✓			✓		
		Head weight (g)	×			×			✓		

DAS in bold font. Cabbage crop type in italic font: *W* white, *R* red, *S* savoy, *P* pointed

*✓* trait scored; *×* trait not scored; BA block A; BB block B

**Fig. 1:**
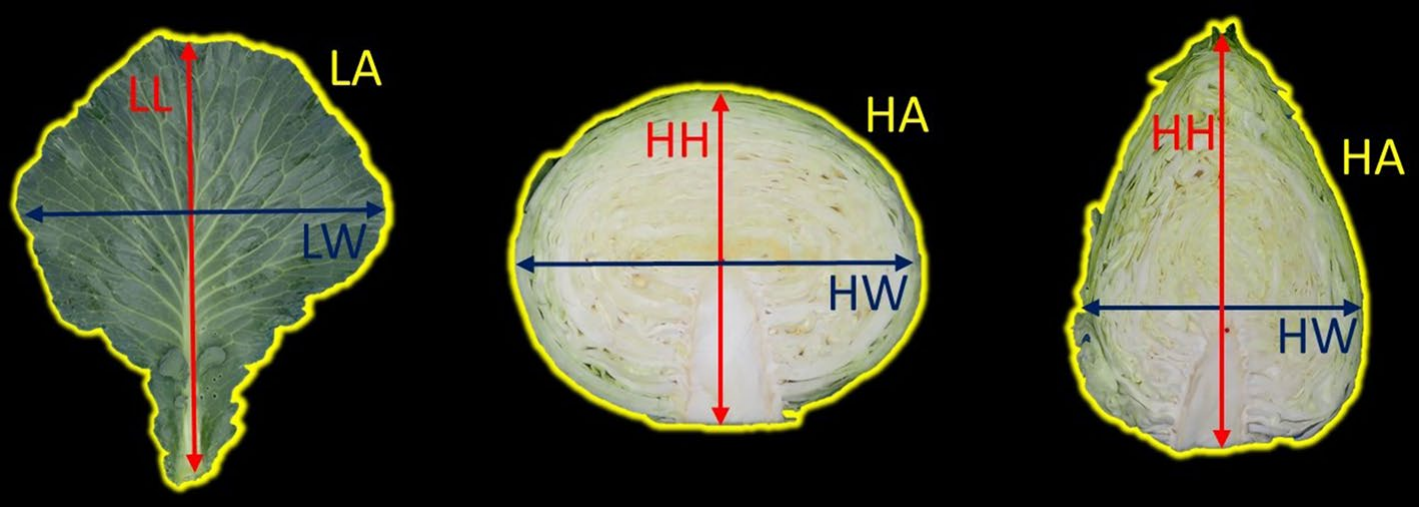
From left to right. Rosette leaf of a white cabbage at HS (left), white cabbage head (centre), and pointed cabbage head (right). Leaf area (LA) limits, leaf length (LL), leaf width (LW), head area (HA) limits, head height (HH), and head width (HW)

After harvesting, rosette leaves and heads sectioned from top to bottom were photographed (Fig. 1). ImageJ v1.52p software (Schneider et al. 2012) was utilized to extract the rosette leaf and head trait measurements from these photographs. An independent phenotypic dataset was created for each trial and included the trait values scored from each cabbage plant per plot.

### Spatial variation correction

It was expected to find local spatial trends within the three field trials. These trends are an effect of the variations in environmental and management conditions. To cope with these spatial trends and correct the phenotypic measurements, the SpATS (Spatial Analysis of Field Trials with Splines) v1.0-15 R package (Velazco et al. 2017; Rodríguez-Álvarez et al. 2018) was utilized. The SpATS model fits a 2D P-splines mixed model to calculate the best linear unbiased estimates (BLUEs) and has been helpful in many studies (Velazco et al. 2017; van Es et al. 2019; Berny et al. 2020; Tsutsumi-Morita et al. 2021). To obtain this adjusted trait estimate for each cabbage accession in a trial, the corresponding phenotypic dataset and the position of each accession plot in the field (in *X* and *Y* coordinate system) were analysed with SpATS and default parameters.

### Statistical analysis

A large amount of rosette leaves and head phenotypic data was collected during the three field trials. A principal component analysis (PCA) was performed to reduce the dimension of the three phenotypic data sets and facilitate the exploration of its variation. To calculate the PCA of the traits, the R function “prcomp” in the “factoextra” v1.0.7 package (Kassambara and Mundt 2020) was utilized. Also, a correlation analysis was performed to study the relationships within and between rosette leaves and head traits. To perform the correlation plots of the traits, the R function “ggpairs” in the “GGally” v2.1.2 package (Schloerke et al. 2021) was used.

### Genome-wide association mapping

The “statgenGWAS” v.1.0.5 R package (van Rossum 2020) was utilized to perform GWAS on all traits. This package utilizes a linear mixed model (LMM) and has been used in several studies (Brzozowsk et al. 2021; Alkemade et al. 2022). With the statgenGWAS package, the genetic relationships between the cabbage accessions were calculated and a kinship matrix was created to correct for population structure. To impute for each year’s SNP dataset the missing SNP dosages, the option Beagle (Browning et al. 2018) from the statgenGWAS package was used. The Beagle model calculates the most likely allele based on the haplotype cluster created by non-missing genotypes. With these parameters and a default threshold of *P* ≤ 0.001 or − 10log(*P*) = 3, a GWAS was performed to each rosette leaf and head trait scored within each trial with two corrections for relatedness between accessions: (1) both kinship + the distance matrix calculated from the PCO axis; (2) only kinship. The Q-Q plot of each GWAS was inspected to evaluate the validity of the correction for population structure. Cheng et al. 2016 estimated that 54.1 Kb is the average distance at which LD decays to half its maximum value based on a set of 43 sequenced cabbage accessions. Based on this, significant SNPs less than 55 kb apart were assumed to indicate the same quantitative trait locus (QTL). QTL “hotspots” were defined as regions with QTLs identified in at least two trials and could include significant SNPs for different traits. The R functions “circos.genomicInitialize”, “circos.track”, “circos.lines”, and “circos.genomicDensity” in the “Circlize” v0.4.15 package (Gu 2014) was used to plot the position of the QTL hotspots on the JZS v2 (Cai et al. 2020) cabbage reference genome. To perform the effect plots of the QTLs, the R function “boxplot” in the “ggplot2” v3.3.5 package (Wickham 2016) was used.

### Re-mapping of hotspots

The Brassicaceae (BRAD) database (http://brassicadb.cn/; accessed 26 April 2021) was utilized to re-map the hotspots identified in the JZS v1 genome to the upgraded JZS v2 (Cai et al. 2020). For this, the complete sequence was extracted from between the two SNPs bordering each hotspot positioned on JZS v1. A minimal sequence length of 2000 base pairs (bp) from the JZS v1 genome was needed to blast it to the JZS v2 genome. When this sequence was less than 2000 bp, the sequence was extended equally in both directions to obtain this minimal length. The genomic region in JZS v2 with the highest match (E-value) was selected as the syntenic region of JZS v1.

### Candidate gene mining

Based on Cheng et al. (2016) average distance at which LD decays to half its maximum value, the QTL hotspots were extended to 55 Kb beyond their original limits and all the *B. oleracea* genes included were extracted. The BRAD database (accessed 11 May 2021) was utilized to BLASTX these *B. oleracea* genes and identify their orthologues in *Arabidopsis thaliana.* The Arabidopsis Information Resource (TAIR) database (http://arabidopsis.org, accessed 11 May 2021) was utilized to annotate the Arabidopsis genes. *B. oleracea* genes of which the ortholog in *A. thaliana* had an annotation related to phytohormones like auxin, cytokinin, gibberellins, and jasmonic acid or leaf development processes like leaf polarity (ad/abaxial) determination and leaf curvature were highlighted as genes of interest.

#### Sampling tissue from heading leaves and RNA extraction

Two hybrid varieties, which are included in the cabbage collection (Supplementary Table 1), a pointed heading (TKI171) and a round heading (TKI028), were utilized to assess their mRNA profile in young heading leaves. Seeds from these accessions were sown in germination trays with sandy soil in the Unifarm greenhouse at Wageningen University & Research (51°59′11″ N latitude, 05°39′52″ E longitude) during the last week of August 2020. Twelve DAS, six seedlings from both accessions were transplanted individually to 2 L pots and placed randomly within the same greenhouse. We followed the development of these accessions throughout the vegetative stage until a leafy head was observable, which for the pointed cabbages was at 63 DAS and round cabbages at 77 DAS. At these time points, respectively, for each cabbage accession, we peeled the wrapping leaves from the leafy heads until a young heading leaf (≈ 1.5 cm) was reached. This leaf was, on average, the 28th (from oldest to younger) for the pointed cabbages and 32nd for the round cabbages. From these leaves we collected tissue. The leaf tissue collected from two plants (randomly selected) from the same cabbage accession was pooled into one sample. In total, six samples were collected: three biological replicates for each cabbage accession. The leaf samples were quickly frozen in liquid nitrogen and stored at − 80 °C. The total RNA was isolated, respectively, from these samples using TRIzol reagent (Invitrogen, USA) according to the manufacturer’s protocol. The quality of the RNA samples was validated to have a high purity (OD260/280 ≥ 2.0 and OD260/230 ≥ 2.0, no degradation, no contamination) and integrity (RIN ≥ 7.0). The RNA of these six samples was shipped to Novogene (UK) for library construction and sequencing with the Illumina NovaSeq 6000 system (Illumina, USA).

#### Messenger-RNA analysis

From Novogene, we obtained 150 bp raw paired-reads. To obtain clean reads, we removed with Trimmomatic v0.39 (Bolger et al. 2014) the reads containing a low-quality and/or Illumina adapters. The parameters for Trimmomatic v0.39 were ILLUMINACLIP = TruSeq3-PE.fa:2:30:10:2, LEADING = 3 TRAILING = 3 MINLEN = 36. These clean reads were mapped to the JZS v2 (Cai et al. 2020) genome utilizing the HISTAT2 2.1.0 (Kim et al*.* 2019) software with –dta and -no-softclip as parameters. These mapped reads were compared to the coding DNA sequences (CDS) of the JZS v2 genome (http://brassicadb.cn/) to estimate the read counts for each gene. For this, we utilized StringTie 2.1.4. (Pertea et al. 2015) software with -e and -G as parameters and the Python script “prepDE.py” also included in StringTie software. The expression levels of all genes are presented as transcripts per million (TPM).

## Results

### Principal coordinate analysis (PCO) of marker data

**Fig. 2:**
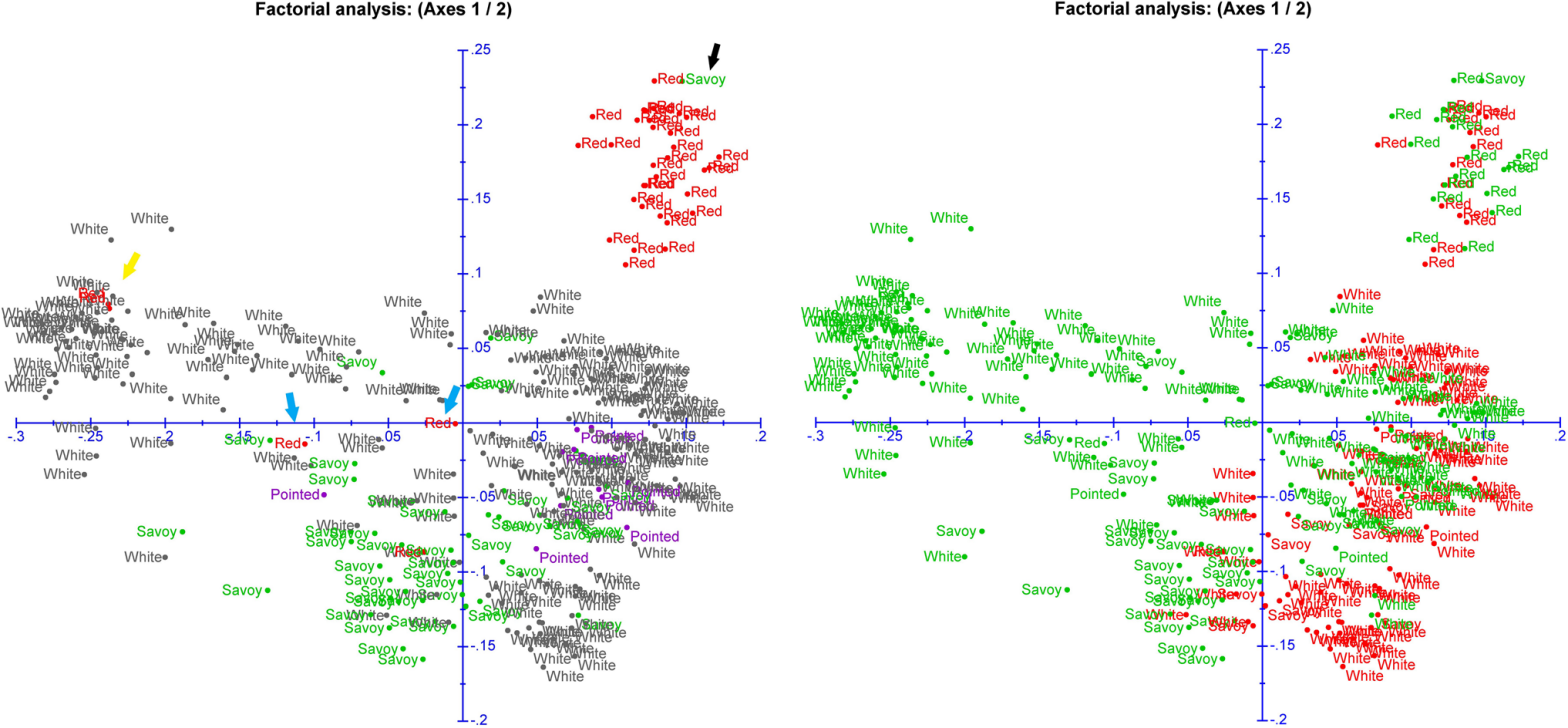
PCO plot (PC1 = 11.95%; PC2 = 5.90%) of accessions included in 2017. Left: Coloured by legend; red cabbages in red, white cabbages in black, savoy cabbages in green and pointed cabbages in purple. The yellow arrow points to two red cabbages with low pigmentation, the blue arrows indicate red cabbages with a pointed head shape, and the black arrow indicates a savoy cabbage with reddish leaves. Right: Coloured by source; F1 hybrids in red and gene bank accessions in green

The cabbage collection utilized in this study included accessions representing different crop types obtained from multiple sources (open-pollinated accessions from gene bank collections and modern F1 hybrids from companies) and collected from different geographical regions. Cai et al. (2022) present a phylogenic analysis of this cabbage collection. In this study, the number of cabbage accessions included in each trial varied (Supplementary Table S1) and as a consequence the SNP set for each trial also varied in number after filtering for a genotyping rate ≥ 80% and MAF ≥ 2.5%. The SNP filtering resulted in the identification of 10,712, 12,803, and 10,112 SNPs over the nine physical chromosomes of the JZS v1 genome, and 3307, 3830, and 3081 over “chromosome zero”, respectively, for 2017, 2018, and 2019 SNP datasets. Most of these SNPs occur in the three data sets. These SNP datasets were utilized to assess the genetic diversity among the cabbage accessions included in each trial. Overall, the PCO plots of 2017 (Fig. 2), 2018 (Supplementary Fig. S1), and 2019 (Supplementary Fig. S2) show similar patterns. The first two principal components show three clusters of accessions (Fig. 2, Supplementary Fig. S1 and S2). The cluster at the left consists almost exclusively of white cabbages coming mostly from the Balkan Peninsula (Bulgaria, Greece, Macedonia, and Turkey). The cluster at the top right includes a dense group of red cabbages. The cluster at the bottom right comprises partly overlapping groups of white, pointed, and savoy cabbages. The more sparsely populated area in the middle includes white, pointed, savoy, and red cabbages. On closer inspection, the first two principal components show that most of the modern hybrid’s cluster at the right, while the genebank accessions are distributed over the whole range from left to right (Fig. 2, Supplementary Fig. S1 and S2). Few accessions dissociate from their peers (Fig. 2, Supplementary Fig. S1 and S2). Most of these accessions show intermediate phenotypes, like red cabbages (Supplementary Fig. S3) with low red pigmentation, or a pointed cabbage and a savoy cabbage (Supplementary Fig. S4) with red (-dish) leaves.

### Phenotypic assessment

The adjusted trait estimates for each cabbage accession obtained with SpATS v1.0-15 (Rodríguez-Álvarez et al. 2018) were used in all further analyses. Descriptive statistics were calculated for the common traits with all the accessions included in each trial (Table 3) and with only the 139 common accessions (Supplementary Table S5), and the trial-specific traits for all accessions (Supplementary Table S6). For the area, length, and width of rosette leaves the white, red, savoy, and pointed accessions scored values within the same range. This was not the case for the head traits area, width, and height, where the pointed accessions were significantly larger and red significantly smaller (Table 3 and Supplementary Table S5). The PCAs for phenotypic traits (Supplementary Fig. S5a, S5b and S5c) also show that red cabbages have the smaller heads, mainly grouping in the quadrants opposite to the direction of the vectors of the head area, head width, and head height, and opposite to the areas containing the white, savoy, and pointed cabbage accessions.

**Table 3:** Descriptive statistics of the phenotypic values scored at heading stage for leaf and head traits

All cabbage accessions								
Trait	Year	Sub-morphotype	*N*	Mean ± S.D	C.V.	Overall mean	C.V.	LSD group
Leaf area	2017	White	200	889.36 ± 324.69	0.37	905.59 ± 321.42	0.35	a
		Red	40	959.99 ± 274.62	0.29			a
		Savoy	40	947.31 ± 368.63	0.39			a
		Pointed	11	850.63 ± 216.21	0.25			a
	2018	White	122	590.40 ± 125.74	0.21	563.03 ± 131.19	0.23	a
		Red	27	498.89 ± 104.53	0.21			b
		Savoy	24	503.46 ± 133.69	0.27			a
		Pointed	7	647.52 ± 147.83	0.23			a
	2019	White	153	823.67 ± 242.37	0.29	837.80 ± 236.66	0.28	a
		Red	43	885.49 ± 184.13	0.21			a
		Savoy	39	801.79 ± 246.64	0.31			a
		Pointed	11	892.02 ± 294.28	0.33			a
Leaf Length	2017	White	200	38.15 ± 8.62	0.23	38.42 ± 8.35	0.22	a
		Red	40	39.96 ± 6.51	0.16			a
		Savoy	40	38.83 ± 9.28	0.24			a
		Pointed	11	36.18 ± 5.15	0.14			a
	2018	White	122	34.56 ± 4.56	0.13	33.87 ± 5.04	0.15	a
		Red	27	35.12 ± 4.12	0.12			a
		Savoy	24	29.57 ± 5.91	0.20			b
		Pointed	7	35.31 ± 6.30	0.18			a
	2019	White	153	39.68 ± 8.03	0.20	40.80 ± 8.14	0.20	b
		Red	43	46.09 ± 6.13	0.13			a
		Savoy	39	39.92 ± 8.32	0.21			b
		Pointed	11	38.79 ± 8.61	0.22			b
Leaf Width	2017	White	200	33.03 ± 6.70	0.20	33.31 ± 6.54	0.20	a
		Red	40	34.25 ± 5.89	0.17			a
		Savoy	40	33.98 ± 6.95	0.20			a
		Pointed	11	32.47 ± 4.08	0.13			a
	2018	White	122	26.98 ± 3.84	0.14	26.23 ± 3.79	0.14	a
		Red	27	24.14 ± 2.82	0.12			a
		Savoy	24	25.13 ± 3.21	0.13			a
		Pointed	7	27.85 ± 3.82	0.14			a
	2019	White	153	32.80 ± 5.27	0.16	32.79 ± 5.03	0.15	a
		Red	43	33.65 ± 4.21	0.13			a
		Savoy	39	31.57 ± 4.51	0.14			a
		Pointed	11	33.65 ± 5.90	0.18			a
Leaf Ratio	2017	White	200	1.22 ± 0.29	0.24	1.21 ± 0.26	0.21	a
		Red	40	1.16 ± 0.20	0.17			a
		Savoy	40	1.19 ± 0.14	0.12			a
		Pointed	11	1.15 ± 0.10	0.09			a
	2018	White	122	1.28 ± 0.24	0.19	1.29 ± 0.24	0.19	b
		Red	27	1.44 ± 0.18	0.13			a
		Savoy	24	1.20 ± 0.19	0.16			b
		Pointed	7	1.26 ± 0.21	0.17			b
	2019	White	153	1.22 ± 0.20	0.16	1.25 ± 0.20	0.16	a
		Red	43	1.37 ± 0.19	0.14			a
		Savoy	39	1.26 ± 0.16	0.13			a
		Pointed	11	1.16 ± 0.19	0.16			a
Head Area	2017	White	166	355.38 ± 75.93	0.21	347.87 ± 74.41	0.21	a
		Red	40	298.06 ± 51.22	0.17			b
		Savoy	32	349.48 ± 63.12	0.18			a
		Pointed	11	411.01 ± 65.99	0.16			a
	2018	White	105	390.10 ± 142.44	0.37	367.88 ± 150.39	0.41	b
		Red	27	213.98 ± 77.90	0.36			c
		Savoy	24	389.73 ± 126.80	0.33			b
		Pointed	7	559.56 ± 95.08	0.17			a
	2019	White	133	232.35 ± 73.53	0.32	206.95 ± 81.35	0.39	a
		Red	42	122.49 ± 57.79	0.47			c
		Savoy	34	193.10 ± 61.98	0.32			b
		Pointed	9	277.92 ± 51.70	0.19			a
Head Width	2017	White	166	22.31 ± 3.20	0.14	21.56 ± 3.37	0.16	a
		Red	40	18.42 ± 2.69	0.15			b
		Savoy	32	21.60 ± 2.88	0.13			a
		Pointed	11	21.47 ± 3.11	0.14			a
	2018	White	105	24.51 ± 5.72	0.23	23.60 ± 6.23	0.26	b
		Red	27	16.75 ± 4.10	0.24			c
		Savoy	24	25.20 ± 4.85	0.19			ab
		Pointed	7	31.31 ± 2.92	0.09			a
	2019	White	133	18.46 ± 4.06	0.22	16.74 ± 4.55	0.27	a
		Red	42	11.64 ± 3.45	0.30			c
		Savoy	34	16.23 ± 3.03	0.19			b
		Pointed	9	17.04 ± 2.39	0.14			ab
Head Height	2017	White	166	19.96 ± 2.60	0.13	20.39 ± 2.84	0.14	b
		Red	40	20.07 ± 2.02	0.10			b
		Savoy	32	20.81 ± 2.46	0.12			b
		Pointed	11	26.70 ± 2.52	0.09			a
	2018	White	105	21.23 ± 4.57	0.22	20.81 ± 4.82	0.23	b
		Red	27	16.72 ± 2.67	0.16			c
		Savoy	24	21.71 ± 4.92	0.23			b
		Pointed	7	27.41 ± 3.23	0.12			a
	2019	White	133	15.66 ± 2.65	0.17	15.39 ± 3.18	0.21	b
		Red	42	13.33 ± 1.99	0.15			c
		Savoy	34	14.79 ± 2.81	0.19			bc
		Pointed	9	23.27 ± 3.34	0.14			a
Head Ratio	2017	White	166	0.86 ± 0.13	0.15	0.92 ± 0.18	0.20	d
		Red	40	1.08 ± 0.16	0.15			b
		Savoy	32	0.94 ± 0.13	0.14			c
		Pointed	11	1.27 ± 0.24	0.19			a
	2018	White	105	0.88 ± 0.13	0.15	0.92 ± 0.14	0.15	b
		Red	27	1.05 ± 0.14	0.13			a
		Savoy	24	0.93 ± 0.13	0.14			b
		Pointed	7	0.93 ± 0.12	0.13			ab
	2019	White	133	0.87 ± 0.15	0.17	0.96 ± 0.22	0.23	c
		Red	42	1.16 ± 0.23	0.20			b
		Savoy	34	0.93 ± 0.16	0.17			c
		Pointed	9	1.37 ± 0.29	0.21			a

LSD groups are limited within year and trait

*n* the number of accessions, *SD*—standard deviation, *CV*—coefficient of variation, *LSD* grouping based on least significant difference

**Fig. 3:**
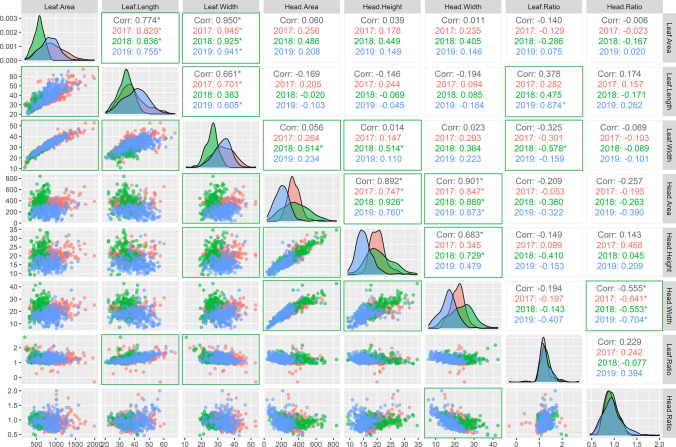
Correlation of common rosette leaf and head traits among all the cabbage accessions tested in all three-year experiments. Upper-part: Correlation coefficients for each year and overall, Diagonal: distribution of averaged trait values; Lower-part: Scatter plot. Correlations marked with * are significant (*P* < 0.05) with a *R*^2^ > 0.5. Green boxes highlight significant correlations

The descriptive statistics (Table 2 and Supplementary Table S5 and S6) and the PCAs (Supplementary Fig. S5a, S5b, and S5c) show phenotypic relationships between and within cabbage rosette leaf and head traits. To visualize these relationships scatter plots, histograms, and correlation coefficients were calculated for the common traits with all the accessions included in each trial (Fig. 3) and the 139 common accessions (Supplementary Fig. S6). Additionally, the complete set of correlations was calculated for all the cabbage accessions including the eight phenotypic traits scored in 2017, 12 in 2018, and 14 in 2019 (Supplementary Fig. S7). The PCA plots (Supplementary Fig. S5a, S5b and S5c), the year-specific correlations, and the overall correlations—correlations values without year distinction—(Fig. 3, Supplementary Fig. S6 and S7) show a high positive correlation (≥ 0.5) between the area, width, and height of the heads except for head width with head height in 2017 (0.345) and 2019 (0.479). The correlations between the area, width, and length of rosette leaves are high except for leaf width with leaf length (0.383) in 2018 (Fig. 3 and Supplementary Fig. S6 and S7). Interestingly, in 2018, correlations between rosette leaf traits and head traits were identified with R-squared (R^2)^ values ranging from − 0.410 till 0.514, while in 2017 from − 0.197 till 0.293 and in 2019 from − 0.407 till 0.262. In 2018, the leaf width showed a slightly higher correlation with both the head area (0.514), and head height (0.514) than the leaf area with the head area (0.486), head height (0.449), and head width (0.405) (Fig. 3 and Supplementary Fig. S6 and S7), while correlation between these head traits and leaf length were low (− 0.020 till 0.085). These latter observations indicate that cabbage plants with larger and wider rosette leaves produced larger heads in 2018. Moreover, the four cabbage crop types (white, pointed, savoy, and red) show similar leaf ratio values, meaning that their rosette leaf shapes are similar. This is not the case for head ratio, as the white and savoy cabbages show lower ratios than the pointed and red cabbages (Table 3 and Supplementary Table S5).

The histograms of the 2017 and 2019 trials (where heads were harvested based on maturity) show lower values with less variation for the head traits area, height, and width and higher values with more variation for the rosette leaf area, length, and width scored at the heading stage than the histograms for the 2018 trial (where heads were harvested in a narrow time window) (Fig. 3 and Supplementary Fig. S6 and S7).

### Association of loci to leaf and head traits in cabbage

**Fig. 4:**
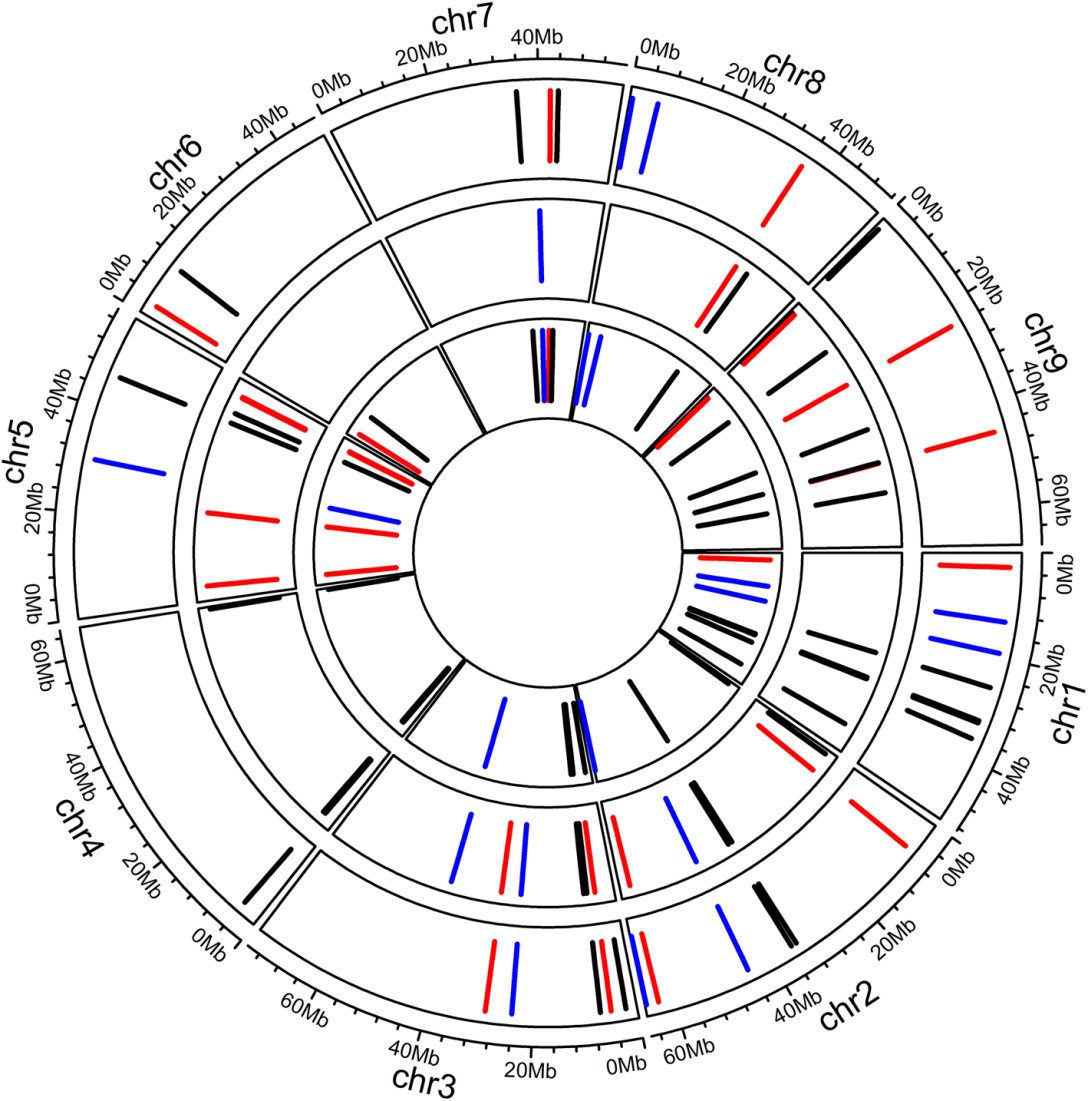
Hotspots distribution over the JZS v2 reference genome. From outer layer to the inner: (a) 2017, (b) 2018, and (c) 2019. Bars in red: hotspots containing only rosette leaf traits; blue: only head traits; and black: both rosette leaf and head traits

In total, 14,019, 16,683, and 13,193 SNPs were identified using JZS v1 genome as reference (including the nine physical chromosomes + chromosome zero), respectively, for 2017, 2018, and 2019 SNP datasets (see Material and Methods). In general, these markers are well distributed over the JZS v1 cabbage genome with some genomic regions covered more densely (Supplementary Fig. S8). These markers are utilized for the GWAS of rosette leaf and head traits scored over the three field trials. In total, the 68 GWAS identified 1703 significant SNP-trait associations (Supplementary Table S7) over the JZS v1 (Liu et al. 2014) genome for the three complete sets of traits (8 for 2017, 12 for 2018, and 14 for 2019) and with both methods to correct the population structure (kinship matrix as default and with or without using the PCO dissimilarity matrix). These 1,703 SNP associations include 1050 SNPs associated to rosette leaf traits and 653 SNPs to head traits (Supplementary Table S7). These 1703 SNPs represent 481 QTLs (see Material and Methods) and 52 of these QTLs are detected in multiple years so are defined as hotspots (see Material and Methods). Supplementary Fig. S9 visualizes, with Manhattan plots, the colocalization of QTLs associated to the same trait (rosette leaf length scored at heading stage) scored in multiple years. The 52 hotspots include 469 SNPs (Supplementary Table S8) and their − 10log(*P*) average (3.76) is significantly higher (*P* value = 1.6 × 10^–12^) than that of the 1234 SNPs (3.53) not included in these hotspots. Fourteen hotspots localize at “chromosome zero” (scaffolds not assigned to a chromosome, see Material and Methods) and 38 are distributed over the nine physical chromosomes (Supplementary Table S8). These 52 hotspots are re-mapped to the updated JZS v2 (Cai et al. 2020) reference genome (see Material and Methods) and all fourteen hotspots on chromosome zero could be mapped to the nine physical cabbage chromosomes (Supplementary Table S9). Adjacent hotspots with a separation of less than 55 kb are considered the same hotspot. Thus, the 52 hotspots identified over the JZS v1 genome result in 50 hotspots over the JZS v2 (Supplementary Table S9). These 50 hotspots include 25 associations with both leaf and head traits, 15 associations with only leaf traits, and 10 with only head traits (Fig. 4 and Supplementary Table S8). The correction for population structure influences the number of hotspots identified (Supplementary Table S8). The kinship matrix is utilized in all analyses to correct the population structure, either with or without additional correction based on the PCO dissimilarity matrix. From the 50 hotspots, 17 are identified both with and without using this dissimilarity matrix, 13 only with and 15 only without using this matrix. Five hotspots are detectable only by combining QTLs from analyses with and without the PCO dissimilarity matrix (Supplementary Table S8). To evaluate the validity of both correction methods for population structure, 34 comparisons of the two Q-Q plots of the two GWAS (one for each correction method) are performed (Supplementary Fig. S10, S11, and S12). In these comparisons, 18 Q-Q plots show a slightly more linear distribution of the SNPs − 10log(*P*) values when both correction methods are utilized and 10 when only the kinship matrix is utilized. Six comparisons show no clear difference between both correction methods.

**Fig. 5:**
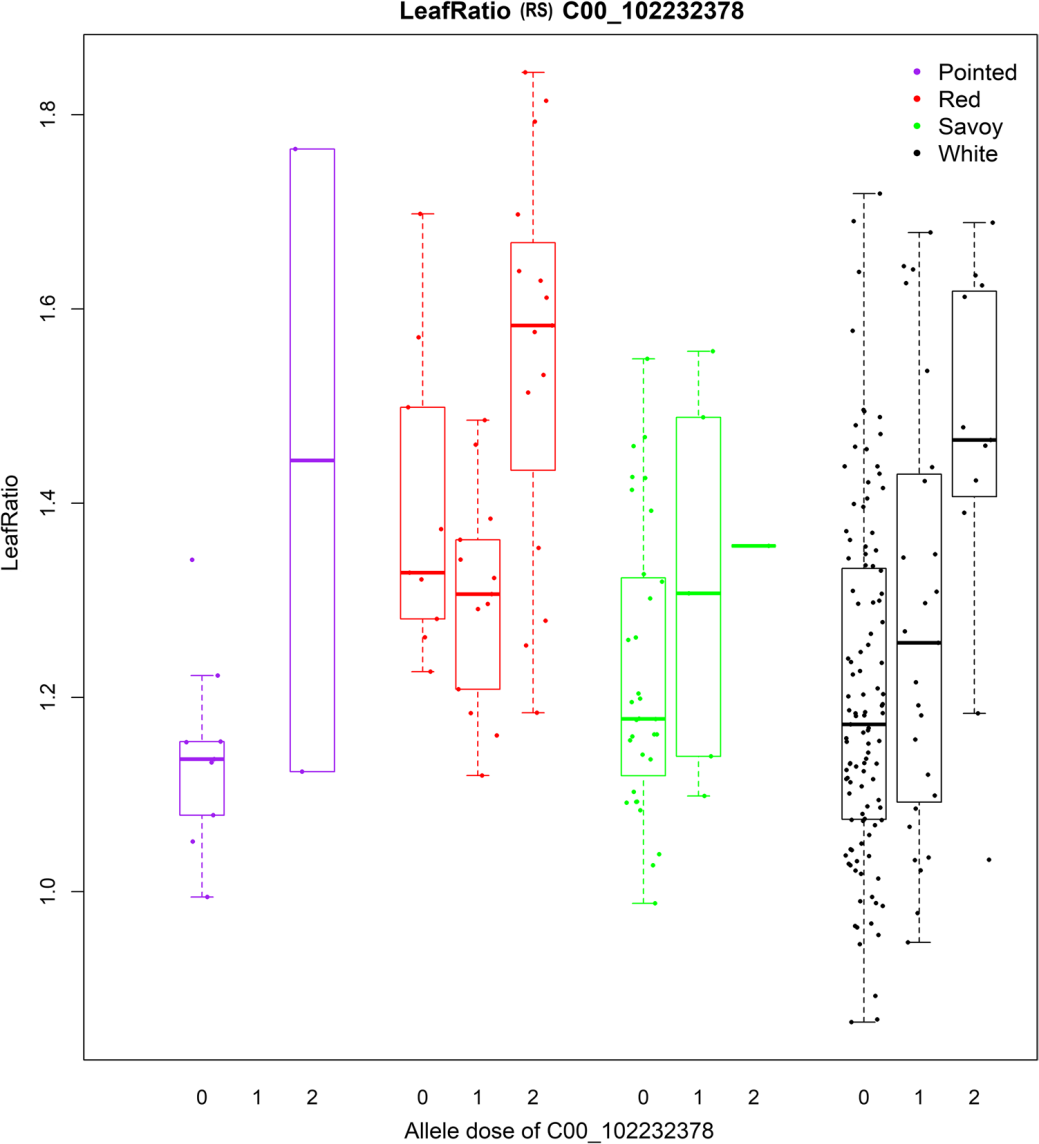
Effect plot of the SNP C00_102232378, included in hotspot #10 (Chr 0 and position 102,232,378 in JZS v1), associated to the leaf ratio scored at rosette stage (RS) in 2019. 0 = homozygous for the reference allele, 2 = homozygous for the alternative allele, 1 = heterozygous

We selected hotspots with allele frequencies > 0.20 and marker-trait associations explaining > 5% of the variation to visualize the effects of the QTLs using boxplots. Hotspot 10 (in JZS v1; hotspot 27 in JZS v2) includes SNP C00_102232378 significantly associated with the 2018 and 2019 Leaf Ratio at rosette stage and 2018 Leaf Ratio at heading stage, and these associations show an allele frequency of approximately 0.25 and explains eight, six, and nine percent, respectively, of the variation (Supplementary Table S8). In these effect plots (Fig. 5, Supplementary Fig. S13a and S13b), the red and white cabbage accessions homozygous for the alternative allele show, overall, higher values in leaf ratio than the accessions homozygous for the reference allele or heterozygous. The differences between the accessions homozygous for the alternative allele and the accessions heterozygous or homozygous for the reference allele is significant. This observation suggests that the reference allele is dominant.

### Candidate gene mining

The border limits of each of the 50 hotspots (JZS v2) are expanded 55 Kb up- and downstream for gene mining (Supplementary Table S10). In total, these expanded regions cover 5.7 Mb of the 561 Mb (1.0%) of the JZS v2 genome and include 763 (Supplementary Table S11) of the 59,064 predicted genes (1.3%). To compare the number of genes included in the JZS v2 hotspots regions to those in JZS v1, we also expanded (55 Kb up- and downstream) the border limits of JZS v1 hotspots (Supplementary Table S12). In total, 629 genes are included in the 38 JZS v1 hotspots located over the nine physical chromosomes (Supplementary Table S12). In JZS v2, the syntenic genomic regions of these 38 hotspots include 638 genes. The 763 cabbage genes identified in JZS v2 hotspots were blasted in the BRAD database (http://brassicadb.cn/; accessed 26 April 2021) to identify their orthologous genes in *A. thaliana* and inspect their annotation in the TAIR database (http://arabidopsis.org, accessed 11 May 2021) (Supplementary Table S11). In total, 591 cabbage genes were found to have an orthologue in Arabidopsis and 550 of these are unique and have an ontology/domain/functional annotation (Supplementary Table S11). Sixteen of these 550 genes match the annotation search terms of a candidate gene (Material and Methods): six are related to phytohormone pathways, eight to leaf development, and two to both sets of annotation terms (Supplementary Table S13). To evaluate the validity of the synteny association between JZS v2 and JZS v1 hotspots, the complete sequences of these genes of interest identified in JZS v2 genome were blasted to JZS v1. For this, we excluded the three genes (BolC02g037390.2J, BolC02g009950.2J and BolC08g038570.2J) identified in JZS v2 hotspots, which are predicted from JZS v1 hotspots localizing in chromosome zero. All the remaining thirteen genes of interest were identified within the syntenic and expanded (55 Kb up- and downstream) regions of the JZS v1 hotspots (Supplementary Table S12 and S13). Additionally, we investigated the distance between the start/stop of these thirteen genes and their associated SNPs. On average, this distance was 12,146 bp, ranging from 1,677 to 36,746 bp (Supplementary Table S13). Interestingly, three of the SNPs associated to Bol025740 (BolC03g043610.2J in JZS v2) and one to Bol018917 (BolC09g026820.2J in JZS v2) are inside the gene sequence. From these four SNPs, only one (C03_16946055) in Bol025740 cause a mutation at a protein level (from arginine to threonine).

To evaluate the participation of the sixteen genes of interest in the leafy head formation process, we analysed their expression in young heading leaves from a round and pointed heading cabbages (see Material and Methods). All genes, except for BolC09g026820.2J, are expressed in young heading leaves of both cabbages (Supplementary Table S14). Moreover, BolC04g067970.2J (in the pointed cabbage) and BolC09g026780.2J (in the round cabbage) are not consistently expressed between replicates. We removed these three genes (BolC04g067970.2J, BolC09g026780.2J and BolC09g026820.2J) from further analysis. The remaining thirteen genes are defined as candidate genes to have a participation in the leafy head formation in cabbage (Table 4).

**Table 4:** Candidate genes putative involved in the leafy head formation in cabbage (*B. oleracea*)

Hotspot	Hotspot identified with traits	Bol name in JZS v2/v1	Other names	Tair or others description
1	Leaf length (HS)	BolC01g004800.2J Bol013627	CER9 and SUD1	Encodes a protein involved in cuticular wax biosynthesis. Lines carrying a recessive mutation in this locus show downward cupped leaves
9	Leaf ratio (RS and HS) Leaf width (RS and HS) Head weight	BolC02g001500.2J Bol005467	LMI1	Encodes a homeodomain leucine zipper class I meristem identity regulator. Acts together with LFY to induce CAL expression. Has a role in leaf morphogenesis
10	Leaf ratio (RS and HS)	BolC02g009950.2J Bol001232	OFP8	Regulates cell elongation by suppressing the expression of Gibberellin 20 oxidase 1
11	Leaf length (HS) Leaf ratio (RS and HS) Head ratio	BolC02g037390.2J	WDS1	Its expression is induced by IAA, ABA, and ethylene
14	Leaf area (RS and HS) Leaf width (HS)	BolC02g059800.2J Bol020633	TCP7	Transcription factor involved in leaf development
18	Leaf ratio (RS) Head area	BolC03g013160.2J Bol025978	SWC6 and SEF	Arabidopsis mutants show serrated leaves
		BolC03g013530.2J Bol026004	CPL4	RNAi suppression mutant lines show epinasty leaves
19	Rosette leaf number Head width	BolC03g014880.2J Bol027883	AGC1-1 and D6PK	Protein kinase involved in polar auxin transport and asymmetrical distribution
21	Leaf area (HS) Leaf length (HS) Leaf width (HS)	BolC03g043570.2 Bol025737	TCP4	Transcription factor regulated by miR319. Involved in the regulation of leaf differentiation
		BolC03g043610.2J Bol025740	CTL02 and TEAR2	Ligase protein that positively regulates CIN-like TCP activity to promote leaf development
26	Leaf area (RS and HS) Leaf length (HS) Leaf width (RS and HS)	BolC05g007470.2J Bol036810	HYL1	Encodes a nuclear dsRNA binding protein involved in mRNA maturation. Mutant show hyponastic leaves and less sensitivity to auxin and cytokinin
42	Leaf length (HS) Head height	BolC08g038570.2J Bol044169	AMP1, COP2, HPT, MFO1, and PT	Encodes glutamate carboxypeptidase. Various alleles show an increased rate of leaf initiation and cytokinin biosynthesis
44	Leaf ratio (RS and HS)	BolC09g002000.2J Bol006070	AXR6, CUL1, ETA1, and ICU13	Encodes a cullin that is a component of SCF ubiquitin ligase complexes involved in mediating responses to auxin and jasmonic acid

*RS*—rosette stage; *HS*—heading stage

## Discussion

### Phenotypic assessment of rosette leaves and heads

The cabbage population consisting of white, pointed, savoy and red accessions, including both modern hybrids and the genetically more diverse gene bank accessions (Cai et al. 2022) forms the basis of this research. White, red, savoy and pointed accessions showed the same range for the rosette leaf traits area, width, length, and ratio. This was not the case for the head traits area, width, height, and ratio. A possible explanation is that cabbage cultivars are usually selected to fit specific head characteristics like size and shape, while less stringent selection is imposed on the size and shape of their rosette leaves. Rosette leaves are important as source organs to produce carbohydrates for the growing leafy heads. Interestingly, in Chinese cabbage, the size and shape of rosette leaves are correlated to those of the leafy head. Chinese leafy heads show, however, a broader range of variation, with overlapping heading leaves like for cabbage heads, but also cylindrical heads with non-overlapping top head leaves (Mao et al. 2014; Sun et al. 2021). Despite the lesser shape variation of cabbage heads, correlations between rosette leaf and head traits were identified.

Overall, the three trials showed differences in rosette leaf and head trait values. Both the 2017 and 2019 trials show higher values with more variation for the rosette leaf traits area, length, and width (measured in rosette and heading stage) than in 2018. The weather reports of the field trials show that the 2018 trial had several periods with higher temperatures, lower precipitation, and higher solar radiation than in the 2017 and 2019 trials. Cabbages like cooler wet growth conditions (fao.org/land–water/), so these differences in climate conditions might be the reason why in 2018 the cabbage plants were less vigorous and thus had smaller rosette leaves. In contrast, in the 2018 trial the leafy head averages had higher values with more variation than in the 2017 and 2019 trials. A possible explanation for this is not so much in the weather, but the difference in the way that the harvesting time was determined. In 2017 and 2019, heads were harvested when estimated mature: however, this resulted systematically in a too early harvest of especially the late (processing and industry) cabbages. In both these 2017 and 2019 trials, the heads were harvested on average 15 days earlier then the fixed harvest of 2018. As a consequence, the cabbage plants in 2018 overall remained longer in the field, and several of the early cabbages could not be harvested as they already cracked. This resulted in on average larger heads. More time to grow implies more time to develop the head characteristics that distinguish each cabbage accession thus, a larger phenotypic variation between accessions.

Determining when the head reaches its maximal potential growth is difficult, especially for industry and processing cabbages, since a tight and compact head can still develop for a longer time without cracking. This probably led to premature harvesting, when selected based on maturity, disrupting both the potential growth of the heads and the development of their specific morphological traits. In contrast, for the early maturing accessions it was easier to determine when the heads were mature since these generally produce relatively small heads (fresh market) and start cracking when harvested too late. This difficulty in assessing correctly head maturity may also explain why only in 2018 a high correlation between rosette leaf and head traits was observed. The underestimation of head maturity in 2017 and 2019 introduced noise and obscured the association between rosette leaf and head traits.

### Genome-wide association of rosette leaves and head traits in cabbage

We refer to QTLs that are detected in several years as QTL hotspots, as these are likely more robust. From these QTL hotspots, we are interested in those associated with both rosette leaves and head traits. We are especially interested in correlations between traits that define shape, as this may point to some common genetic regulation, while correlation between size may reflect differences in vigour between accessions and seasonal variation between years. We see a high correlation between rosette leaf and head traits scored in 2018, which includes shape traits, possibly a result of such common regulatory mechanisms.

Some of our QTL hotspots (in JZS v1 genome) associate with Lv et al. 2014 and Cheng et al. 2016 QTLs (also in JZS v1 genome), which are also associated to the heading trait in cabbage. Hotspots 17, 18, and 19 roughly overlap with two QTLs identified by Lv et al. 2014 at the middle part of chromosome 1, hotspot 21 with a QTL at the beginning of chromosome 2 and hotspot 41 with a QTL at the end of chromosome 6. Hotspot 17 also localizes in a selective sweep identified by Cheng et al. 2016 at chromosome 1, hotspot 50 in a selective sweep at chromosome 9, and hotspot 51 in another selective sweep also at chromosome 9 (Supplementary Table S15). Additionally, hotspot 23 localizes, approximately, 31,000 bp away a selective sweep at chromosome 2.

The hotspots identified in JZS v1 (Liu et al. 2014) cabbage reference genome were re-mapped to the upgraded version JZS v2 (Cai et al. 2020). Interestingly, the 50 hotspots identified in both reference genomes show that all nine *B. oleracea* chromosomes participate, through many QTLs, in controlling both rosette leaf and head traits, and half of these hotspots control traits in both organs. Although the correlation between rosette leaf traits and head traits scored in the 2017 and 2019 trials is lower, some hotspots include associations to both rosette leaf and head traits scored within these years.

All hotspots were identified with SNPs with a − 10log(*P*) value higher than 3.0. The use of a relatively low − 10log(*P*) threshold increases the probability of obtaining false positives. By considering only hotspots including SNPs identified in multiple year trials (with a different cabbage collection subset), we intended to lower the impact of these false associations. This approach excludes the true marker-trait associations identifiable only under the unique environmental conditions occurring in a single trial but instead increases the probability of identifying more robust associations.

### Candidate gene mining

GWAS is a powerful tool to associate genomic regions with phenotypic traits. The regions we identified in this study include many genes, most of which remain unannotated in cabbage. This complicates the identification of the specific genes causing the phenotypic variation in cabbage rosette leaves and heads. Thirteen genes, expressed in the heading leaves of two cabbage accessions, were selected as candidate genes based on their orthologous annotation in *A. thaliana* (found in the TAIR database). As candidates genes, we include those that can induce inward leaf curvature. This is an essential characteristic of heading leaves to overlap each other around the shoot apex and produce a leafy head (Mao et al. 2014). One mechanism that induces leaves to curve is the arrested growth of the marginal regions in comparison to the central region of the leaf (Nath et al. 2003). We hypothesize that this differential growth occurs while the leaf develops, mainly during cell division and cell expansion processes. Within our QTL hotspots, we identified genes for the transcription factors TCP4 (BolC03g043570.2J2) and TCP7 (BolC02g059800.2J) which are involved in the leaf development. In Chinese cabbage, TCP4 regulates cell division along the leaf blade and a cell division arrest in the leaf marginal regions produces differences in head shape (Mao et al. 2014). We also include a gene (BolC03g043610/Bol025740) that encodes a ligase protein that positively regulates CINCINNATA (CIN)-like TCPs activity, which control the morphology and size of leaves (Nath et al. 2003). Interestingly, this gene includes in its sequence a SNP that associates with rosette leaf traits and causes a mutation at a protein level. Additionally, we included a gene (BolC02g009950.2J) involved in regulating the biosynthesis of gibberellin, which has an impact in the cell cycle regulation during cell division.

Leaf curvature also results from a difference in growth rates of the abaxial and adaxial sides (Liu et al. 2011; Sandalio et al. 2016; Li et al. 2019). For this reason, we selected as candidate genes those with a role in leaf adaxial/abaxial polarity determination. Auxin has an essential role in the leaf polarity determination by controlling AUXIN RESPONSE FACTORS ARF3 and ARF4. These transcription factors interact with members of the YABBY family to determine the abaxial side in crosstalk with members of the KANADI family. The studies of Cheng et al. (2016) in *B. rapa* and *B. oleracea* and Liang et al. (2016) in *B. rapa* suggest that auxin response factors participate in the heading trait. Thus, we include as candidates, genes induced by auxin (BolC02g037390.2J) or that mediate responses to auxin (BolC09g002000.2J). Moreover, the localization of auxin gradients specifies the final leaf shape. These gradients are formed by auxin carriers like AUX1 and PIN1. For this reason, we include an auxin transporter (BolC03g014880.2J) gene as candidate. We also selected as candidate a gene (BolC02g001500.2J) member of the HD-ZIP III transcription factors family which determine the adaxial side (Kalve et al. 2014) and was identified in selection signals related to the heading trait in cabbage (Cheng et al. 2016).

We are also interested in genes that affect the shape of the leaves. We selected *B. *oleracea genes orthologous to genes in Arabidopsis for which mutants show downward (BolC01g004800.2J and BolC03g013530.2J) or upward (BolC05g007470.2J) curved leaves, serrated leaves (BolC03g013160.2J) or an increased rate of leaf initiation (BolC08g038570.2J).

The possible involvement of these thirteen genes in the observed leaf and head traits should be studied by inspecting their allelic variation for possible mutations in their coding region or regulatory regions.

One interesting candidate is the *BolC05g007470.2J,* orthologous to the Arabidopsis gene *HYPONASTIC LEAVES 1* (*HYL1*) that plays an essential role in miRNA biogenesis and leaf development. *HYL1* regulates the expression of micro-RNAs miR165/166, miR319a, and miRNA160 that play a role in leaf flattening through the relative activities of adaxial and abaxial identity genes (Liu et al. 2011). The *HYL1* orthologous gene in Chinese cabbage is *LEAFY HEADS* (*BcpLH*). Functional studies in Chinese cabbage suggest that *BcpLH* controls the expression of many miRNAs and coordinates during head formation the direction, extent, and timing of leaf curvature (Ren et al. 2020). We hypothesize that the *HYL1* orthologous gene in cabbage (*B. oleracea*) also plays a role in leaf curvature and head formation.

## Conclusion

The formation of the leafy head is complex and most knowledge of the heading trait in *Brassica* species comes from Chinese cabbage. This study shows that head formation in cabbage and Chinese cabbage share many similarities. In both species, the heading trait is regulated by many QTLs and in both species morphological traits of the rosette leaves are correlated to those of the leafy head. In addition, several candidate genes are detected for both species with roles in ad/abaxial leaf development, leaf proliferation, and/or auxin pathways, processes that previously were associated with leaf curvature and leafy head formation (such as the Arabidopsis orthologous gene *HYL1* and genes related to leaf polarity and development).

## Supplementary Information

Below is the link to the electronic supplementary material.

Supplementary Figure S1 PCO plot (PC1 = 12.99 %, PC2 = 5.68%) of accessions included in 2018. Left) Coloured by variety: white in grey, red in red, savoy in green and pointed in purple. Right) Coloured by source: breeders’ material in green and gene bank material in red (JPG 2110 kb) Supplementary Figure S1 PCO plot (PC1 = 12.99 %, PC2 = 5.68%) of accessions included in 2018. Left) Coloured by variety: white in grey, red in red, savoy in green and pointed in purple. Right) Coloured by source: breeders’ material in green and gene bank material in red (JPG 2110 kb)

Supplementary Figure S2 PCO plot (PC1 = 11.74%; PC2 = 7.04%) of accessions included in 2019. Left) Coloured by variety: white in grey, red in red, savoy in green and pointed in purple. Right) Coloured by source: breeders’ material in green and gene bank material in red (JPG 2452 kb) Supplementary Figure S2 PCO plot (PC1 = 11.74%; PC2 = 7.04%) of accessions included in 2019. Left) Coloured by variety: white in grey, red in red, savoy in green and pointed in purple. Right) Coloured by source: breeders’ material in green and gene bank material in red (JPG 2452 kb)

Supplementary Figure S3 From left to right and top to bottom: TKI428, TKI746, TKI777, TKI989 red cabbages (JPG 1384 kb) Supplementary Figure S3 From left to right and top to bottom: TKI428, TKI746, TKI777, TKI989 red cabbages (JPG 1384 kb)

Supplementary Figure S4 TKI645 savoy cabbage (JPG 2923 kb) Supplementary Figure S4 TKI645 savoy cabbage (JPG 2923 kb)

Supplementary Figure S5 PCA of the phenotypic values scored for leaf and head traits (JPG 65625 kb) Supplementary Figure S5 PCA of the phenotypic values scored for leaf and head traits (JPG 65625 kb)

Supplementary file6 (JPG 65625 kb)

Supplementary file7 (JPG 65625 kb)

Supplementary Figure S6 Correlation of common rosette leaf and head traits among the 139 common cabbage accessions tested in all three-year experiments. Upper-part: Correlation coefficients for each year and overall, Diagonal: distribution of averaged trait values; Lower-part: Correlations marked with * are significant (P<0.05) with a R²>0.5 (PNG 7867 kb)

Supplementary Figure S7 Correlation of all rosette leaf and head traits scored in all three-year experiments among all the cabbage accessions tested in each trial. Upper-part: Correlation coefficients for each year and overall, Diagonal: distribution of averaged trait values; Lower-part: Scatter plot. Correlations marked with * are significant (P<0.05) with a R²>0.5 (PNG 8541 kb)

Supplementary Figure S8 Combined presentation of the three SNP datasets distributed over the nine physical chromosomes of JZS v1 (Liu et al., 2014) cabbage reference genome (JPG 151875 kb) Supplementary Figure S8 Combined presentation of the three SNP datasets distributed over the nine physical chromosomes of JZS v1 (Liu et al., 2014) cabbage reference genome (JPG 151875 kb)

Supplementary Figure S9 Manhattan plots (2017, 2018 and 2019) showing the SNPs (in red) significantly associated to the rosette leaf length scored at heading stage and using Kinship to correct for population structure. The red circles highlight the SNPs included in hotspot 18 (JZS v1) and blue circles in hotspot 30 (JZS v1) (JPG 3126 kb) Supplementary Figure S9 Manhattan plots (2017, 2018 and 2019) showing the SNPs (in red) significantly associated to the rosette leaf length scored at heading stage and using Kinship to correct for population structure. The red circles highlight the SNPs included in hotspot 18 (JZS v1) and blue circles in hotspot 30 (JZS v1) (JPG 3126 kb)

Supplementary Figure S10 Comparison of Q-Q plots from 2017 GWAS (PDF 832 kb)

Supplementary Figure S11 Comparison of Q-Q plots from 2018 GWAS (PDF 1241 kb)

Supplementary Figure S12 Comparison of Q-Q plots from 2019 GWAS (PDF 1304 kb)

Supplementary Figure S13 Phenotypic effect plots of selected SNPs included in hotspot #10 (in JZS v1 reference genome); 0 = homozygous for the reference genome, 2 = homozygous for the alternative genome, 1 = heterozygous (TIFF 1617 kb)

Supplementary file16 (TIFF 1628 kb)

Supplementary file17 (XLSX 500 kb)

## Data Availability

The data for this article have been deposited in Figshare: 10.6084/m9.figshare.c.6175330.v1.
