# Supplementary figures and images for: Genetic dissection of morphological variation in rosette leaves and leafy heads in cabbage (Brassica oleracea var. capitata)
Source: Theor Appl Genet. 2022 Sep 3;135(10):3611–28. doi: 10.1007/s00122-022-04205-w (PMC9519658; doi:10.1007/s00122-022-04205-w)

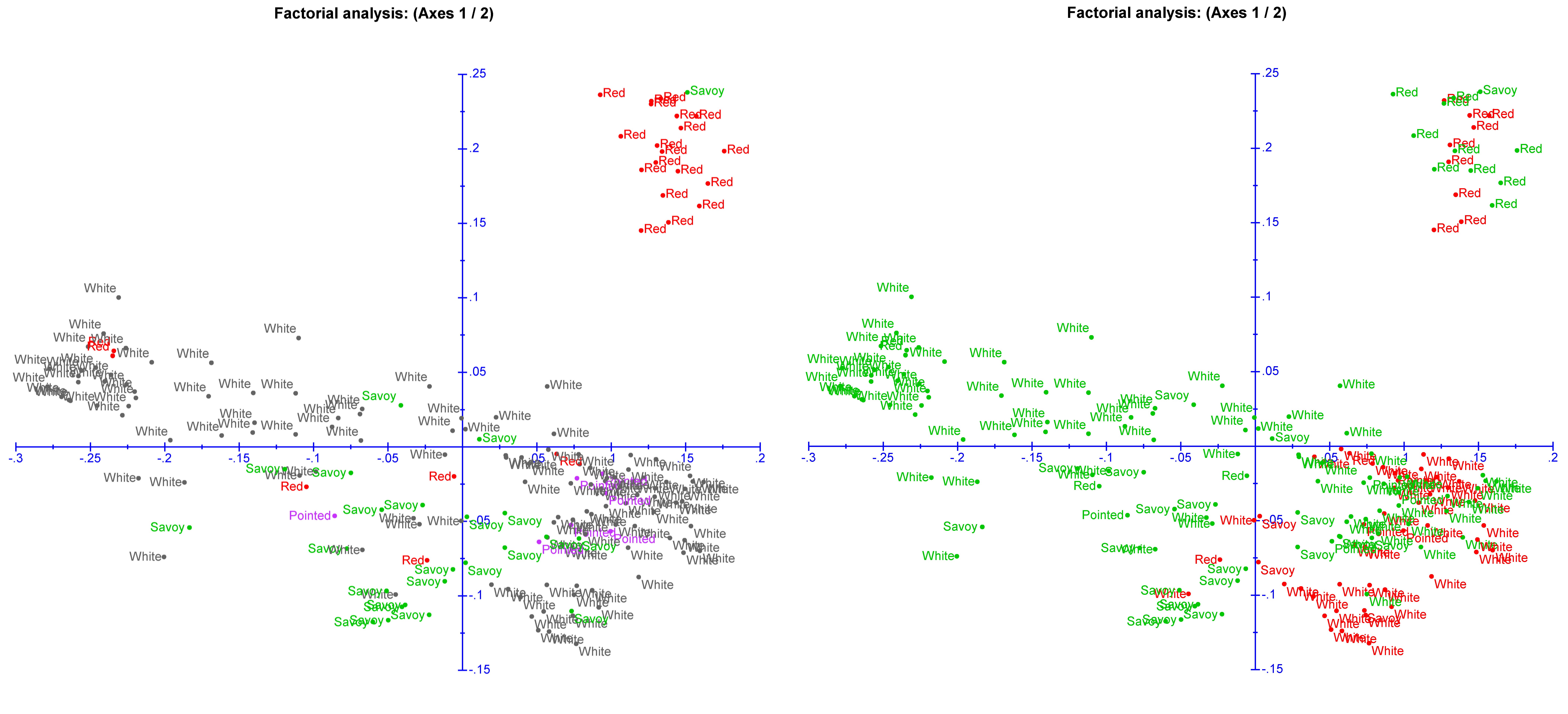

Supplement: Supplementary file 1 — Supplementary Figure S1 PCO plot (PC1 = 12.99 %, PC2 = 5.68%) of accessions included in 2018. Left) Coloured by variety: white in grey, red in red, savoy in green and pointed in purple. Right) Coloured by source: breeders’ material in green and gene bank material in red (JPG 2110 kb) [file 122_2022_4205_MOESM1_ESM.jpg]

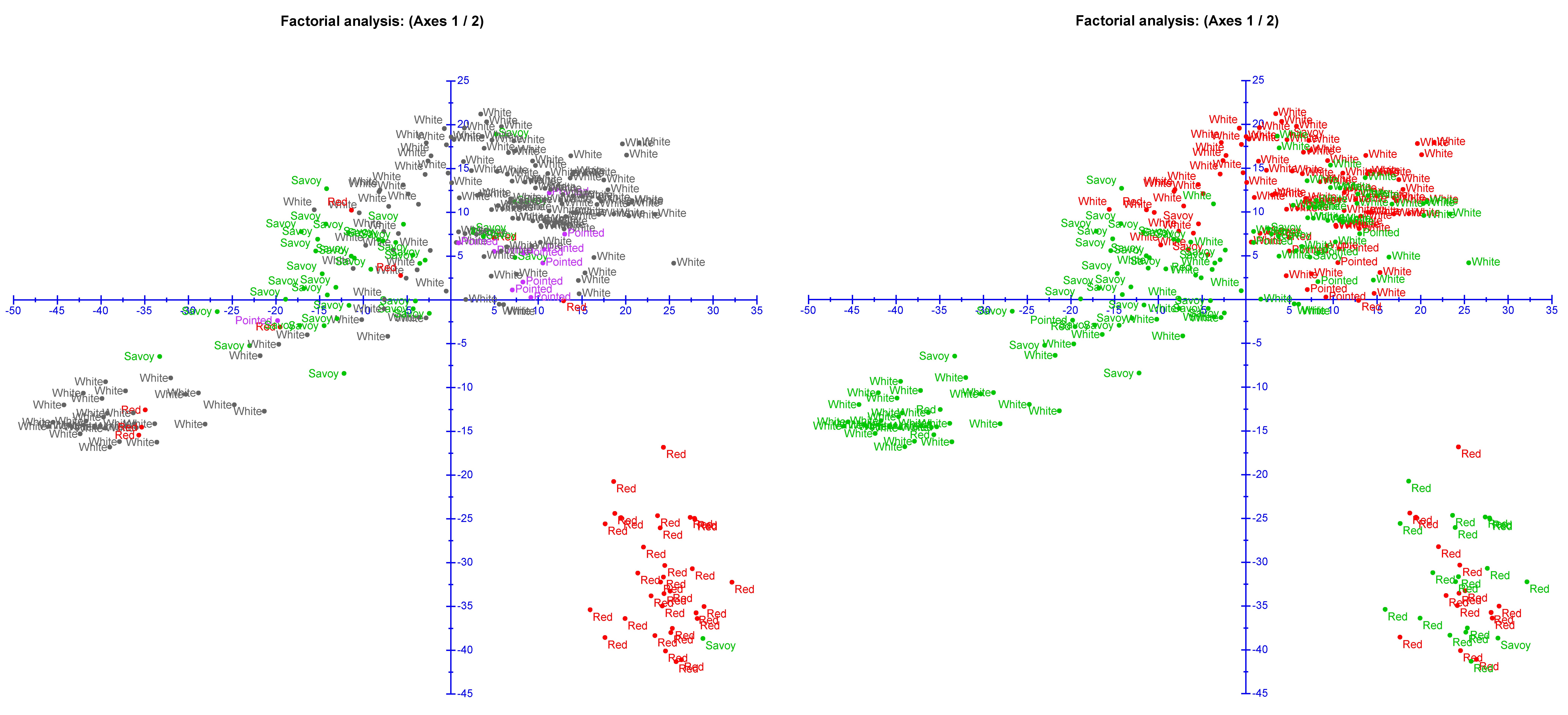

Supplement: Supplementary file 2 — Supplementary Figure S2 PCO plot (PC1 = 11.74%; PC2 = 7.04%) of accessions included in 2019. Left) Coloured by variety: white in grey, red in red, savoy in green and pointed in purple. Right) Coloured by source: breeders’ material in green and gene bank material in red (JPG 2452 kb) [file 122_2022_4205_MOESM2_ESM.jpg]

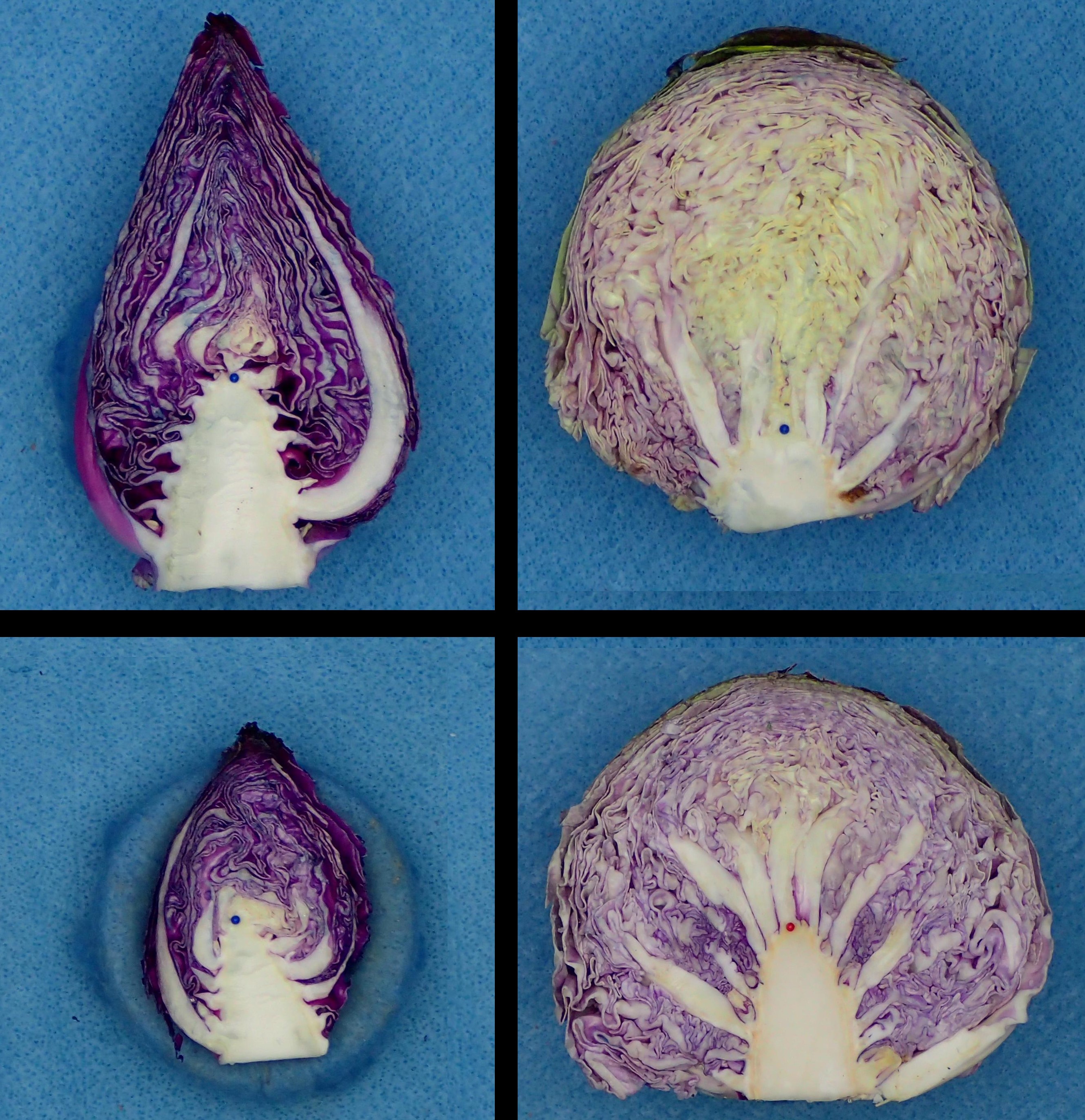

Supplement: Supplementary file 3 — Supplementary Figure S3 From left to right and top to bottom: TKI428, TKI746, TKI777, TKI989 red cabbages (JPG 1384 kb) [file 122_2022_4205_MOESM3_ESM.jpg]

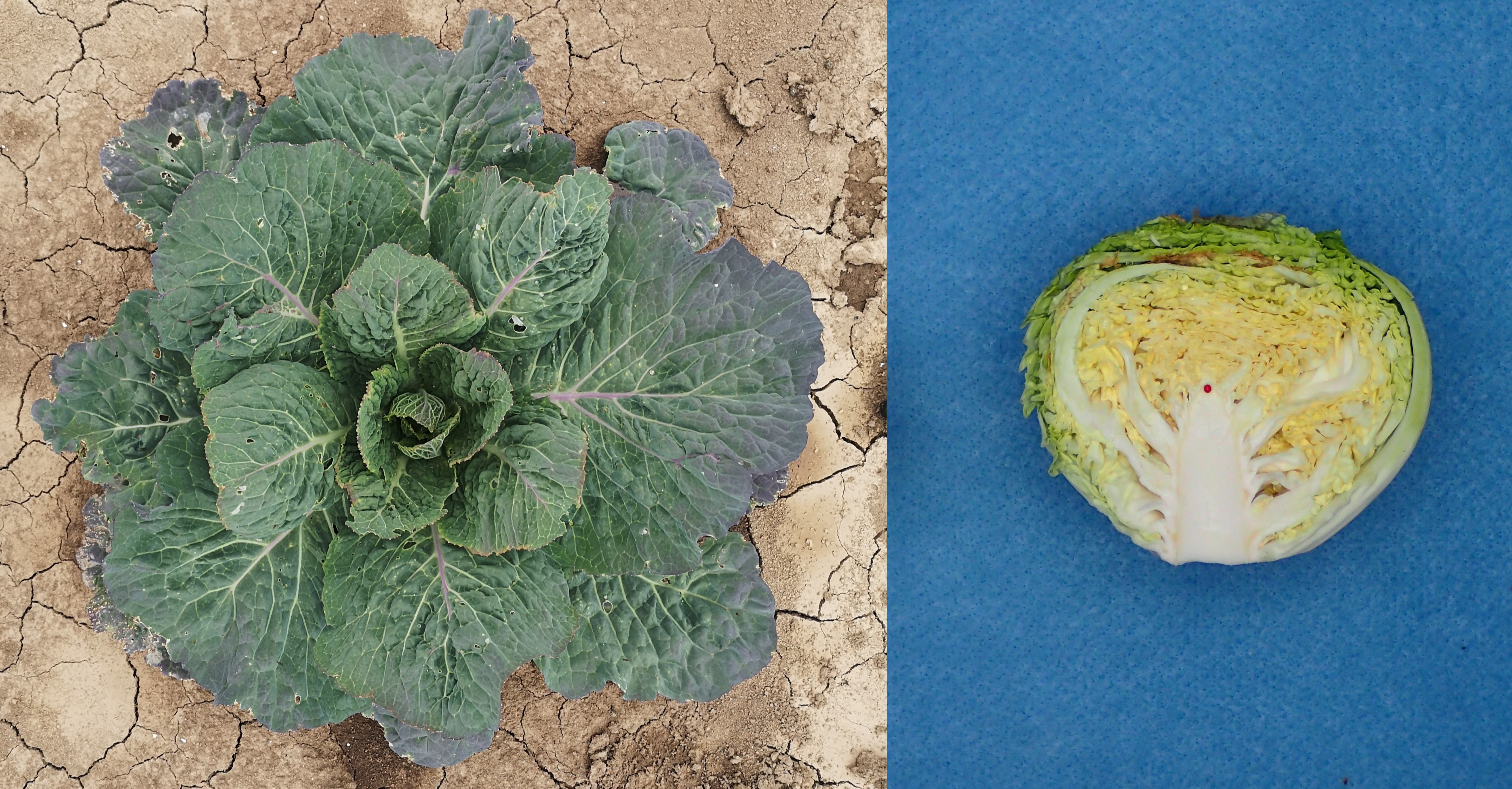

Supplement: Supplementary file 4 — Supplementary Figure S4 TKI645 savoy cabbage (JPG 2923 kb) [file 122_2022_4205_MOESM4_ESM.jpg]

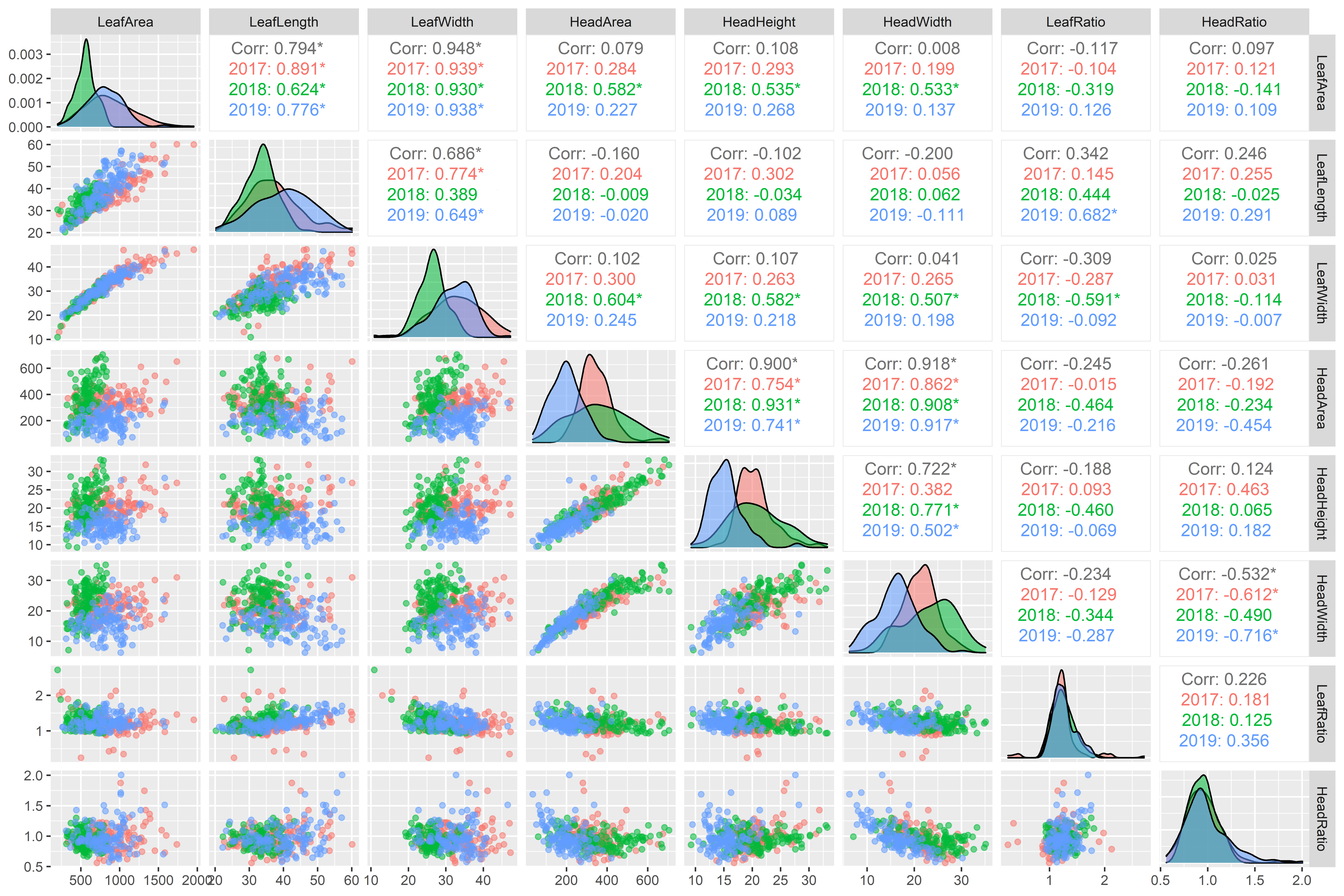

Supplement: Supplementary file 8 — Supplementary Figure S6 Correlation of common rosette leaf and head traits among the 139 common cabbage accessions tested in all three-year experiments. Upper-part: Correlation coefficients for each year and overall, Diagonal: distribution of averaged trait values; Lower-part: Correlations marked with * are significant (P<0.05) with a R²>0.5 (PNG 7867 kb) [file 122_2022_4205_MOESM8_ESM.png]

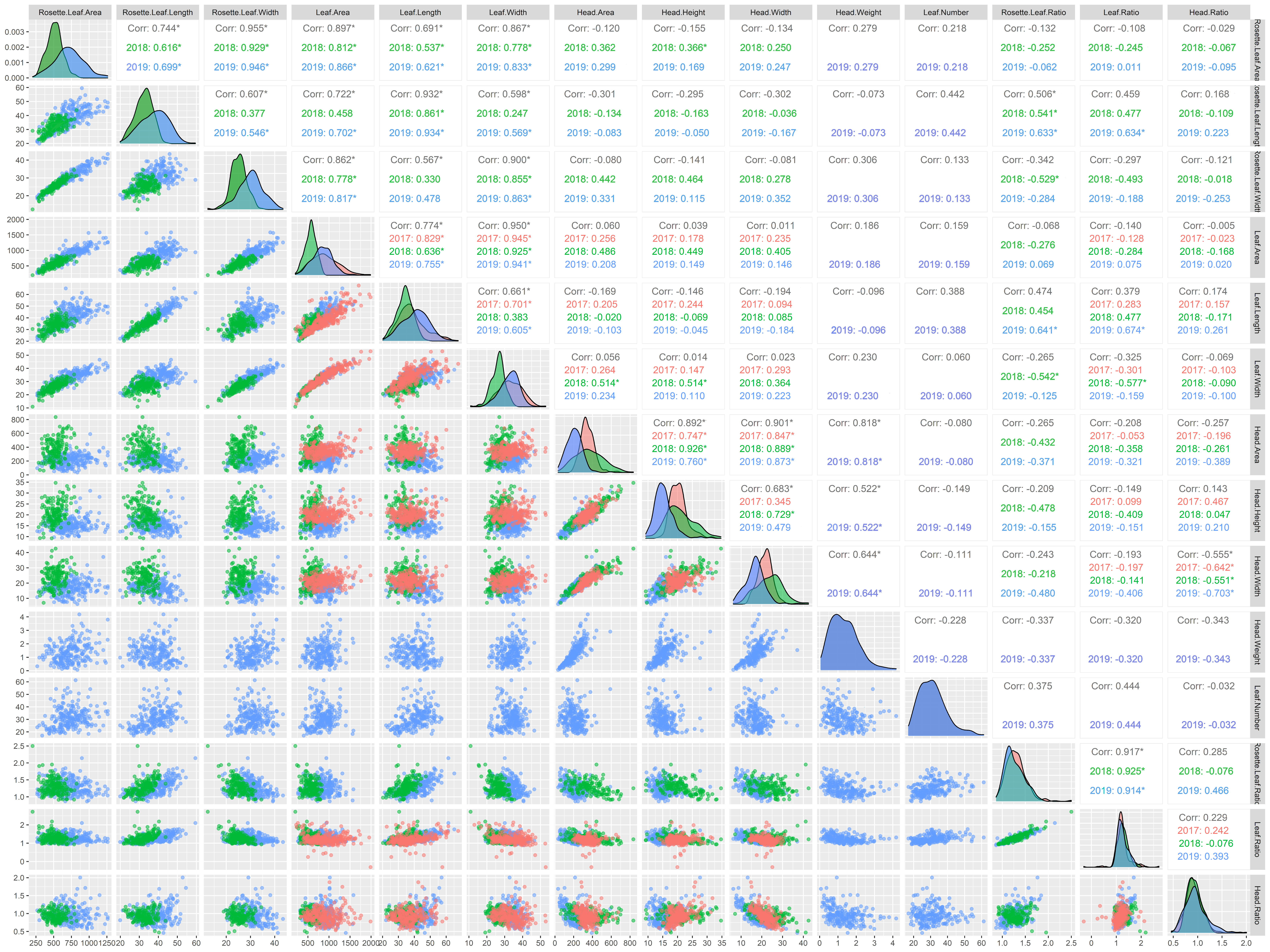

Supplement: Supplementary file 9 — Supplementary Figure S7 Correlation of all rosette leaf and head traits scored in all three-year experiments among all the cabbage accessions tested in each trial. Upper-part: Correlation coefficients for each year and overall, Diagonal: distribution of averaged trait values; Lower-part: Scatter plot. Correlations marked with * are significant (P<0.05) with a R²>0.5 (PNG 8541 kb) [file 122_2022_4205_MOESM9_ESM.png]

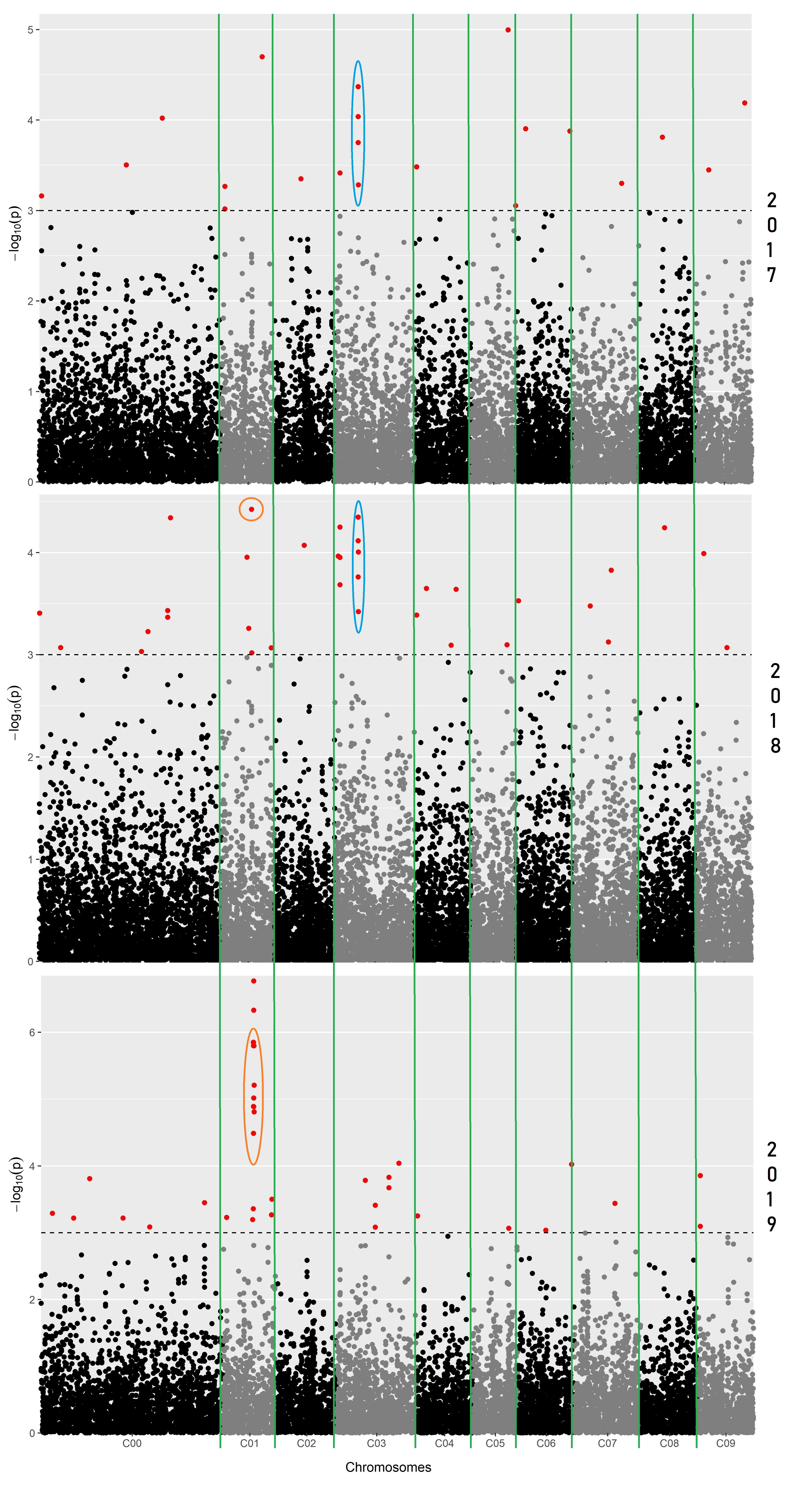

Supplement: Supplementary file 11 — Supplementary Figure S9 Manhattan plots (2017, 2018 and 2019) showing the SNPs (in red) significantly associated to the rosette leaf length scored at heading stage and using Kinship to correct for population structure. The red circles highlight the SNPs included in hotspot 18 (JZS v1) and blue circles in hotspot 30 (JZS v1) (JPG 3126 kb) [file 122_2022_4205_MOESM11_ESM.jpg]

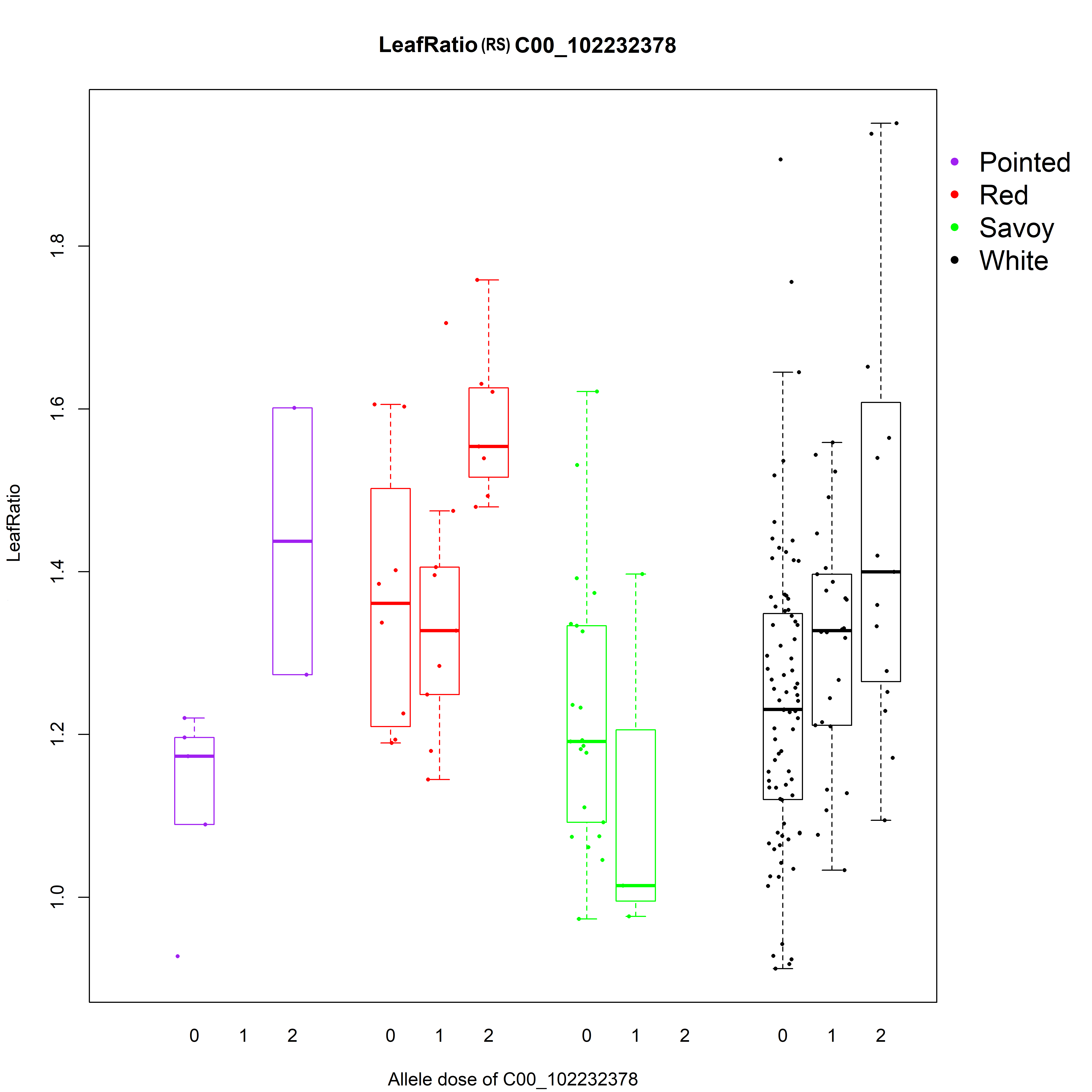

Supplement: Supplementary file 15 — Supplementary Figure S13 Phenotypic effect plots of selected SNPs included in hotspot #10 (in JZS v1 reference genome); 0 = homozygous for the reference genome, 2 = homozygous for the alternative genome, 1 = heterozygous (TIFF 1617 kb) [file 122_2022_4205_MOESM15_ESM.tiff]

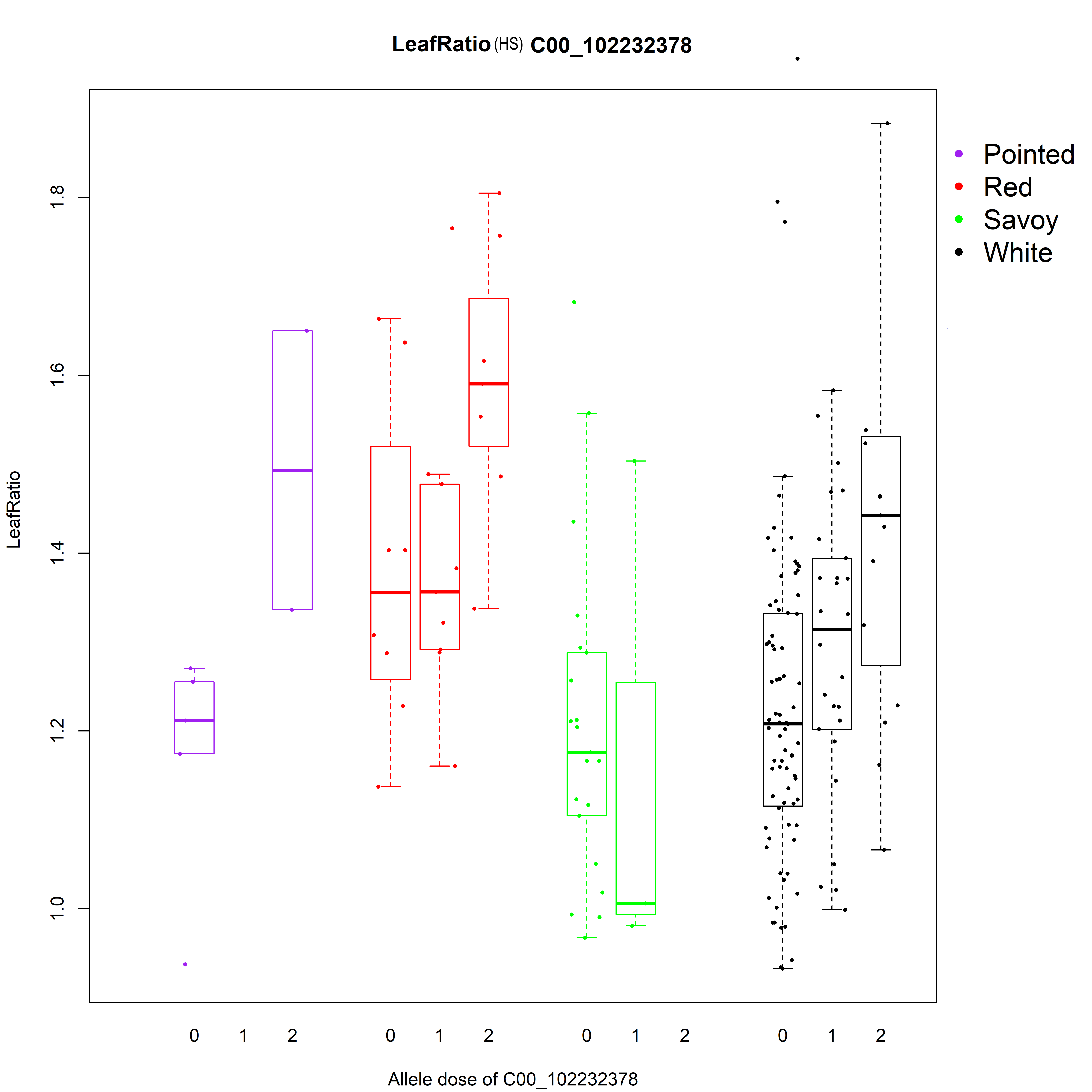

Supplement: Supplementary file 16 — Supplementary file16 (TIFF 1628 kb) [file 122_2022_4205_MOESM16_ESM.tiff]
