# Supplementary material for: Genetic dissection of morphological variation in rosette leaves and leafy heads in cabbage (Brassica oleracea var. capitata)
Source: Theor Appl Genet. 2022 Sep 3;135(10):3611–28. doi: 10.1007/s00122-022-04205-w (PMC9519658; doi:10.1007/s00122-022-04205-w)

Leaf Area (heading stage). Population structure correction based on Principal Coordinates and Kinship (left), or Kinship only (right).

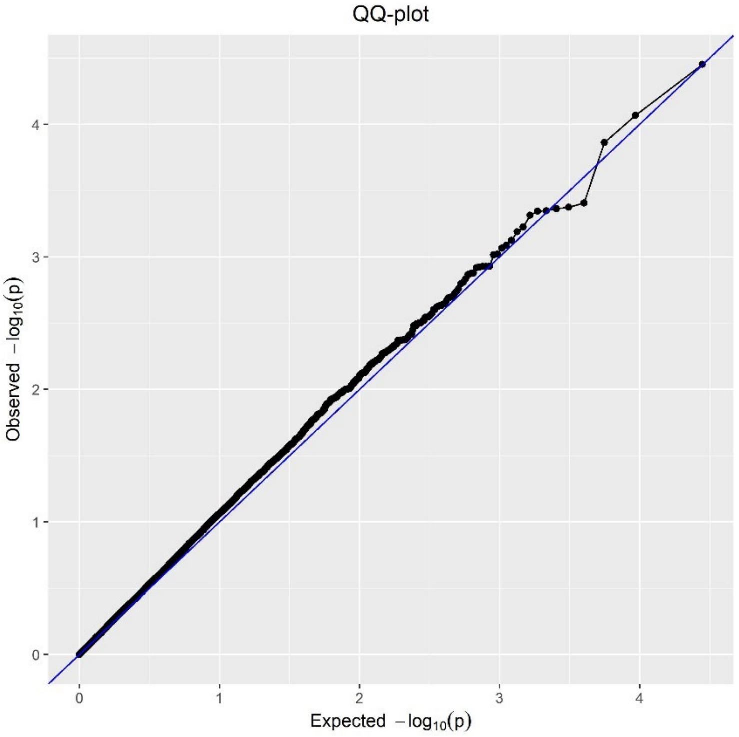

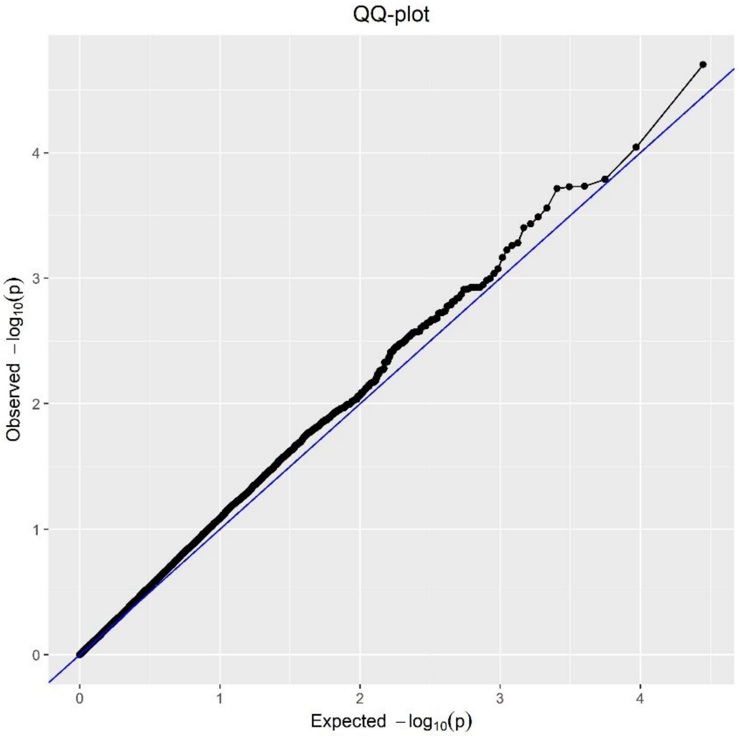

Leaf Width (heading stage). Population structure correction based on Principal Coordinates and Kinship (left), or Kinship only (right).

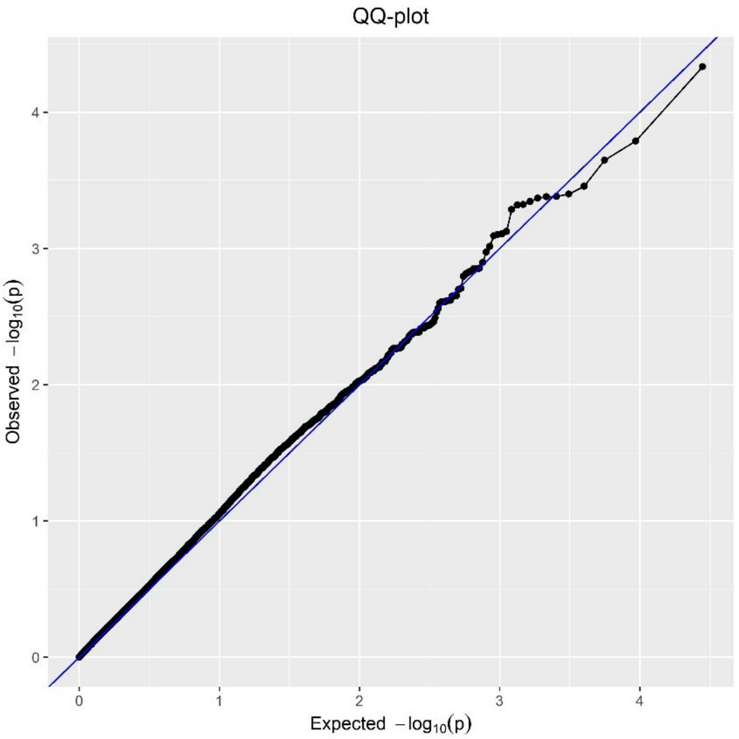

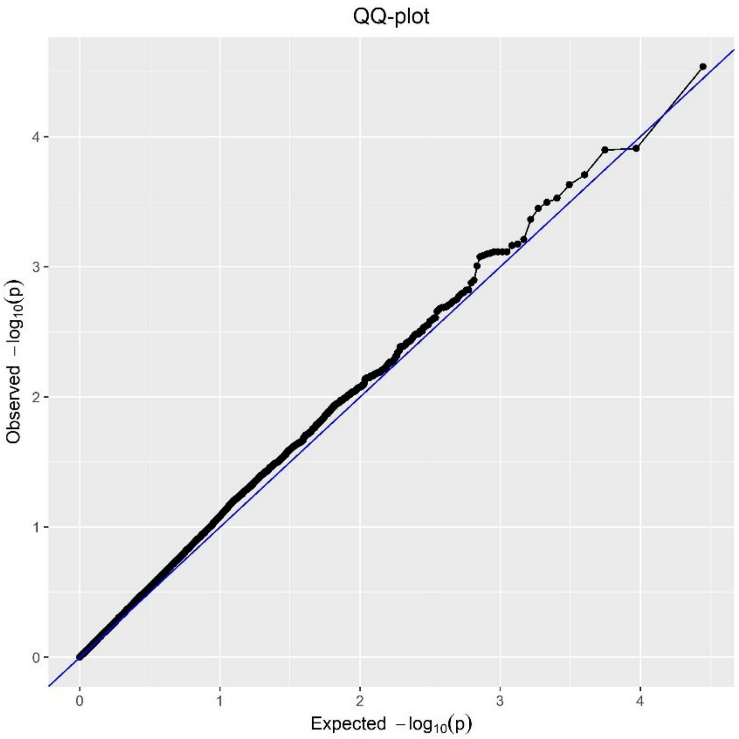

Leaf Length (heading stage). Population structure correction based on Principal Coordinates and Kinship (left), or Kinship only (right).

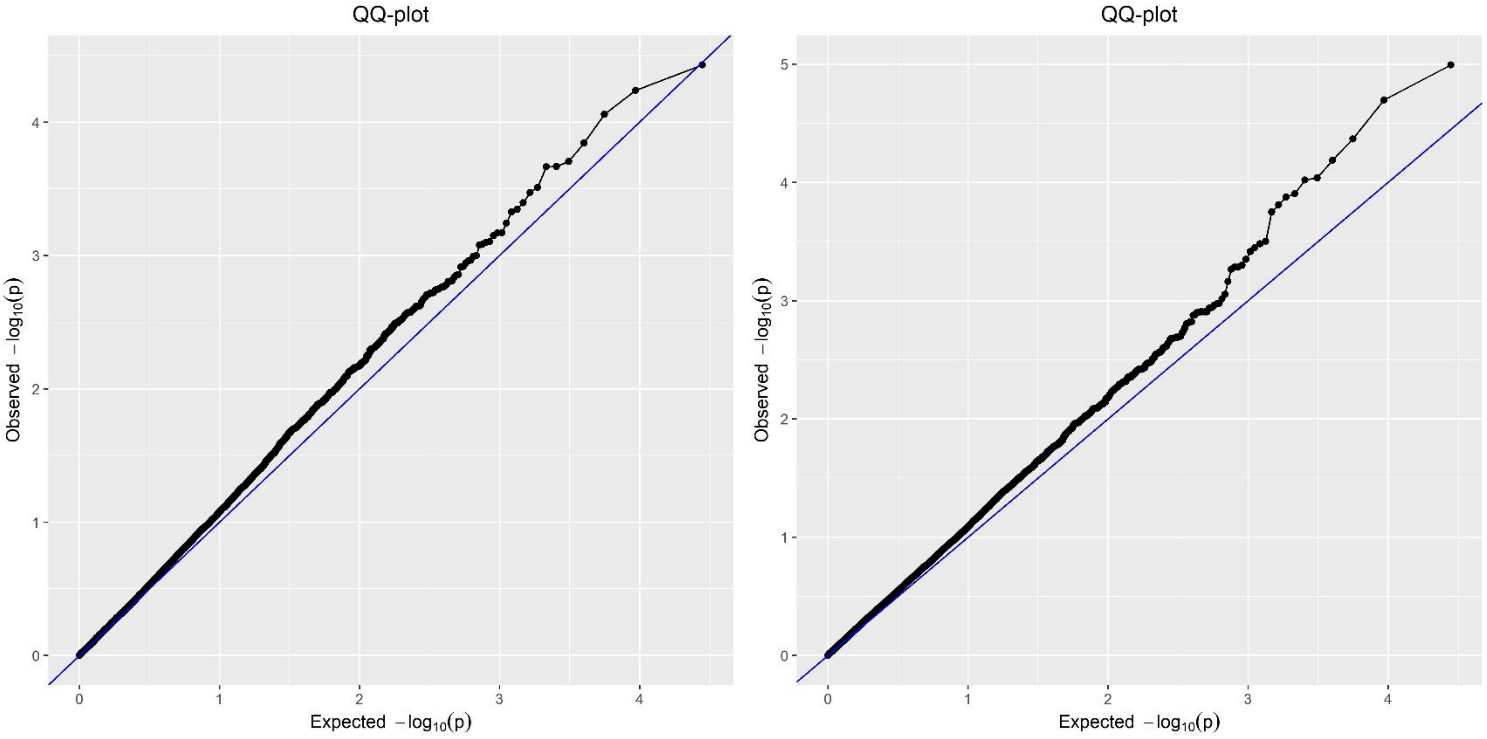

Leaf Ratio (heading stage). Population structure correction based on Principal Coordinates and Kinship (left), or Kinship only (right).

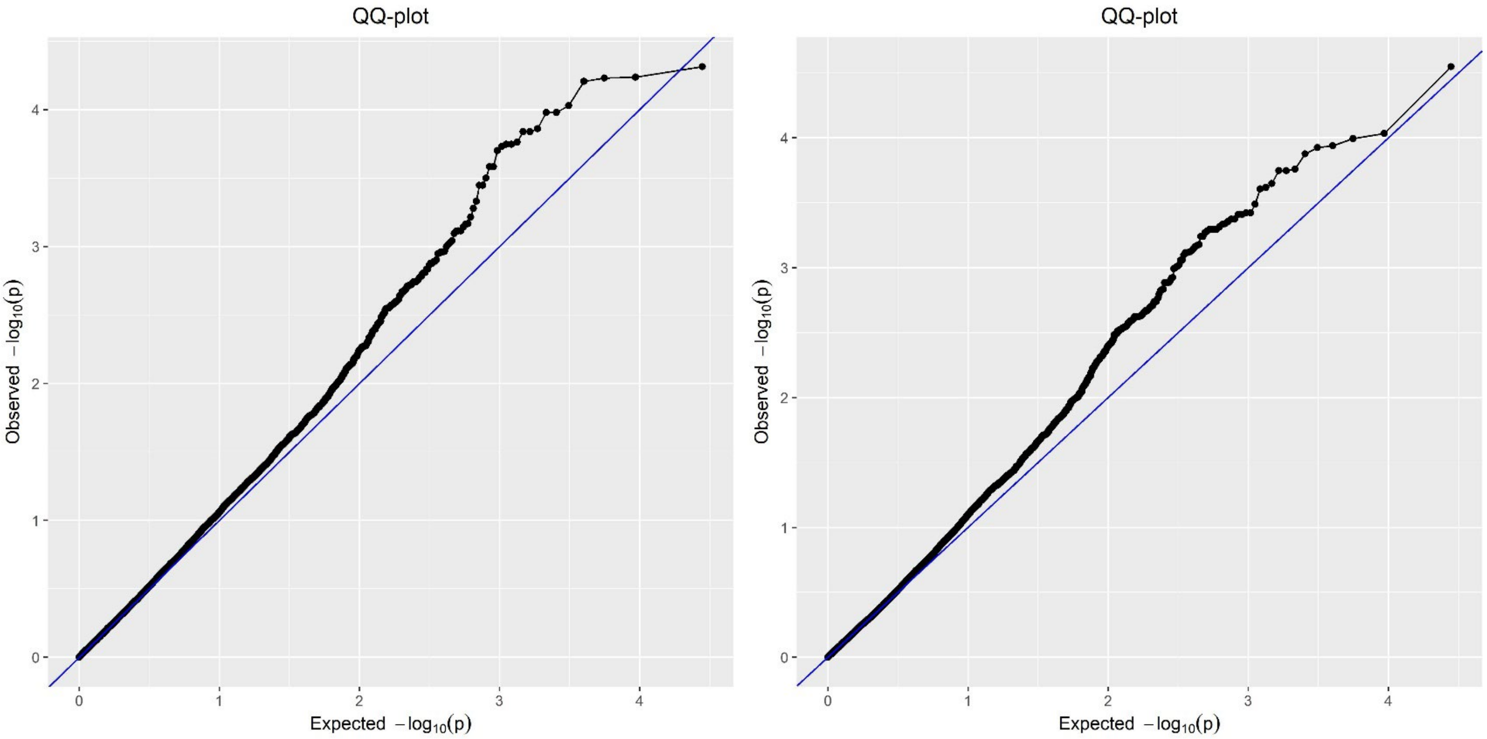

Head Area. Population structure correction based on Principal Coordinates and Kinship (left), or Kinship only (right).

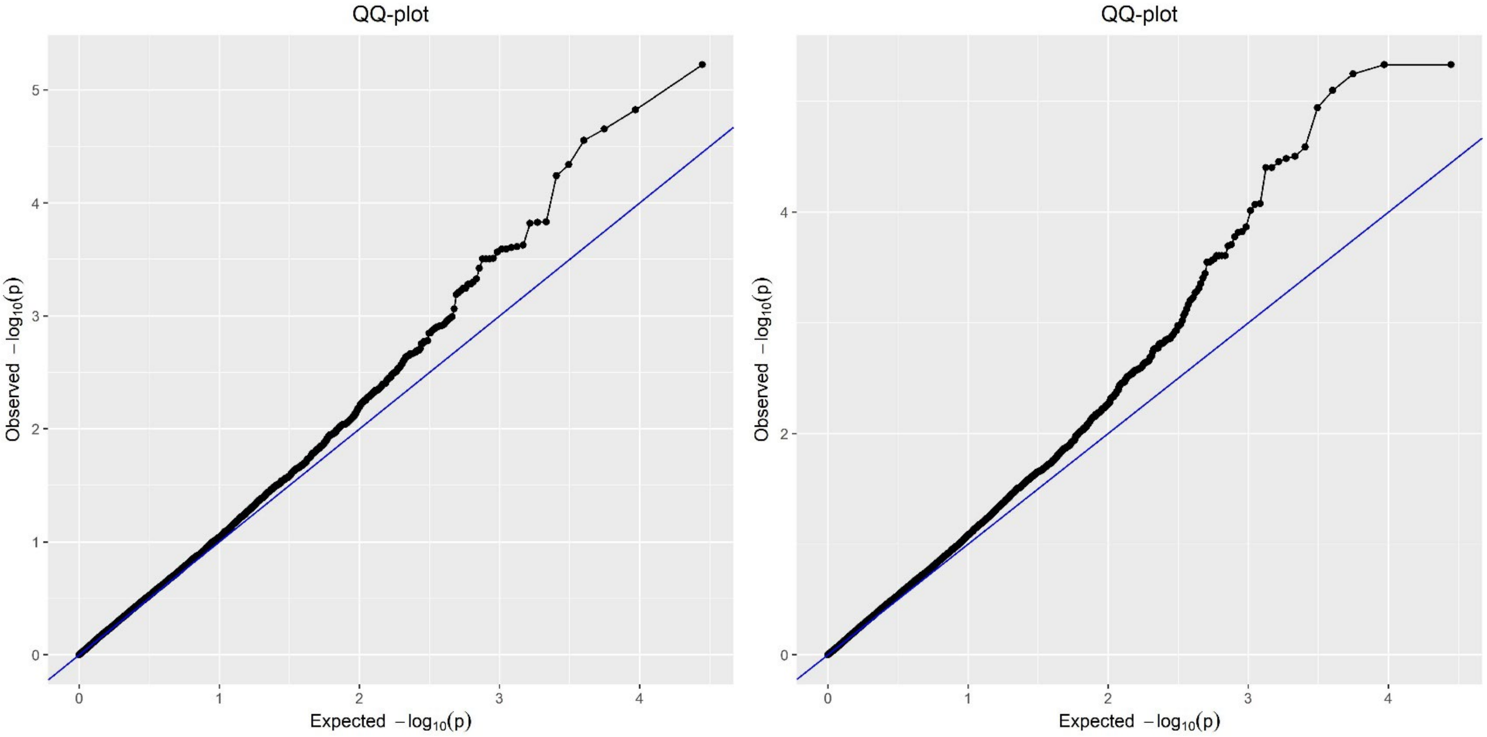

Head Width. Population structure correction based on Principal Coordinates and Kinship (left), or Kinship only (right).

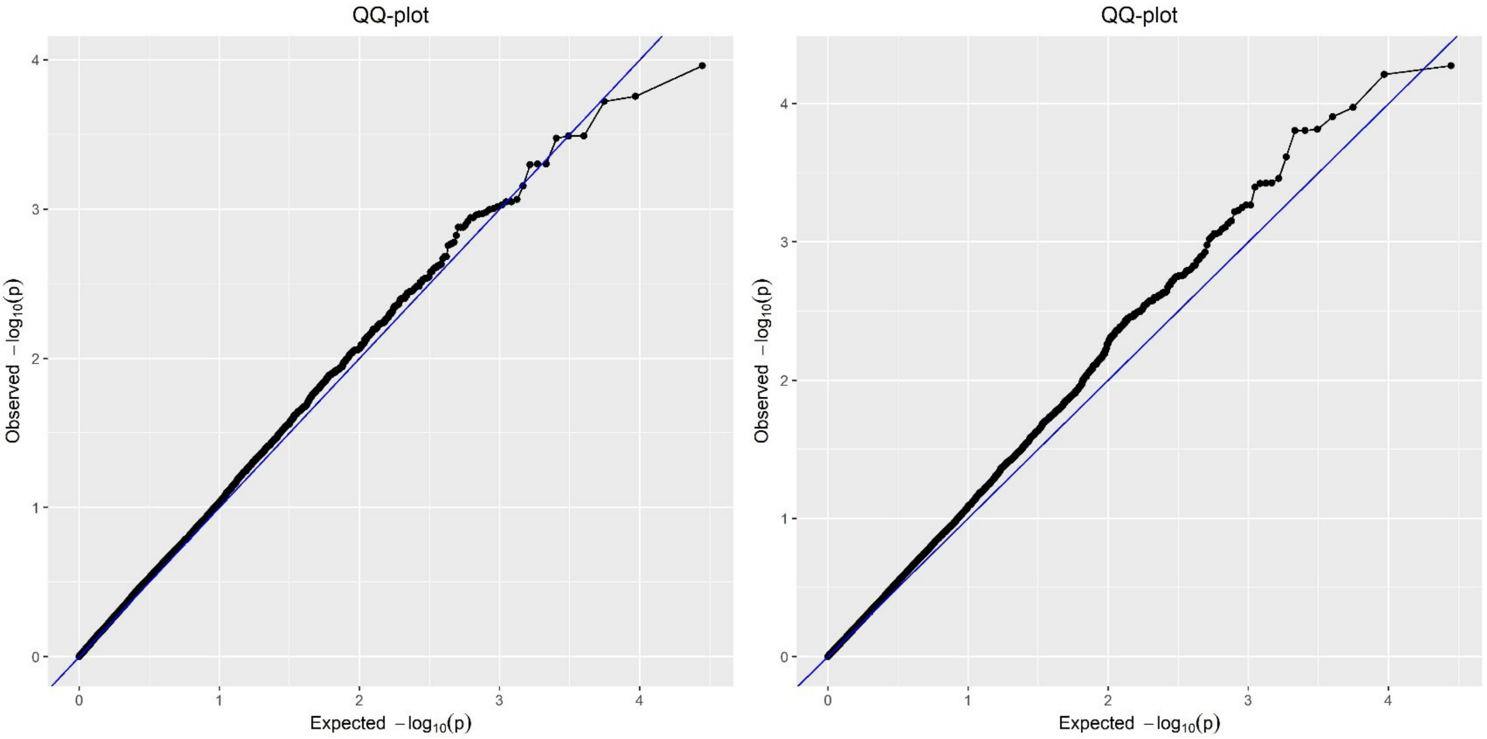

Head Height. Population structure correction based on Principal Coordinates and Kinship (left), or Kinship only (right).

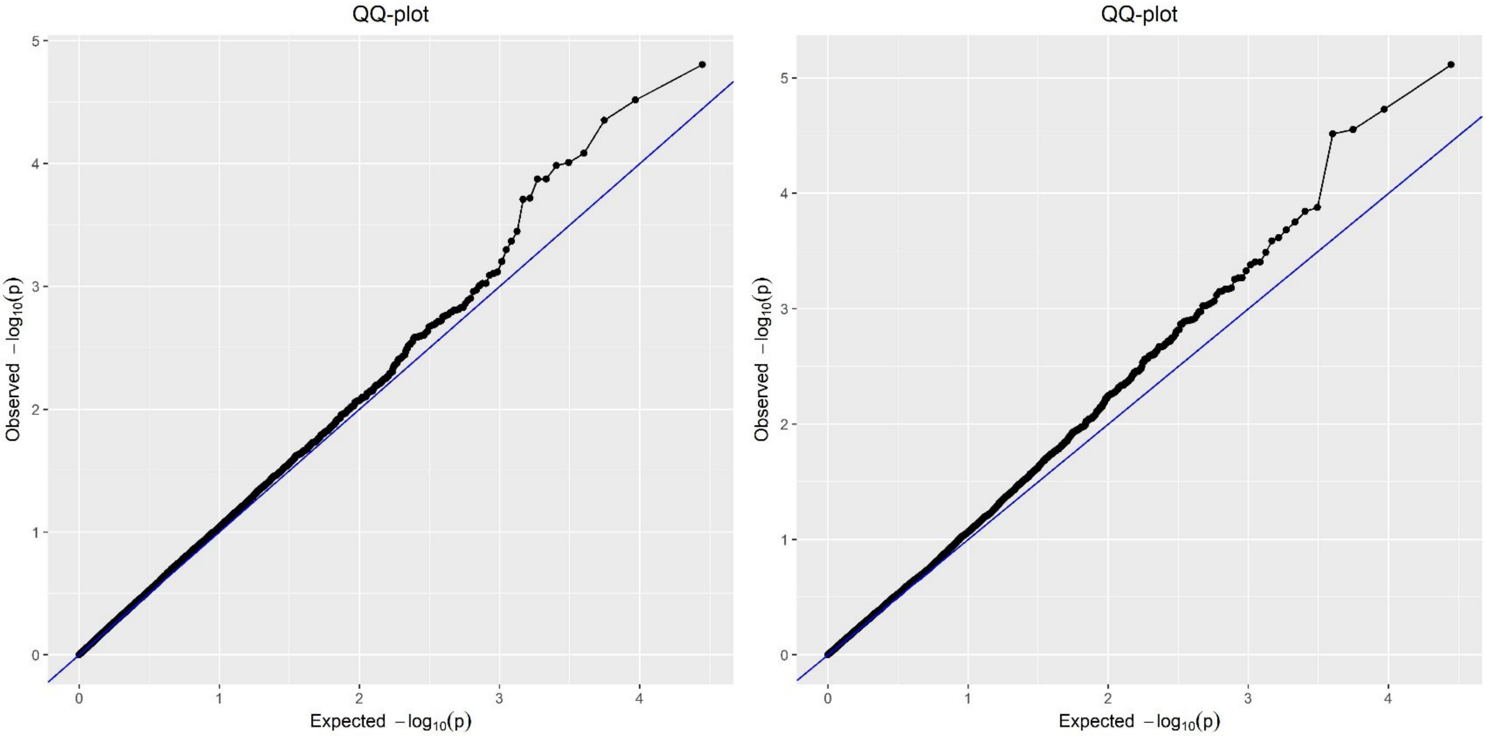

Head Ratio. Population structure correction based on Principal Coordinates and Kinship (left), or Kinship only (right).

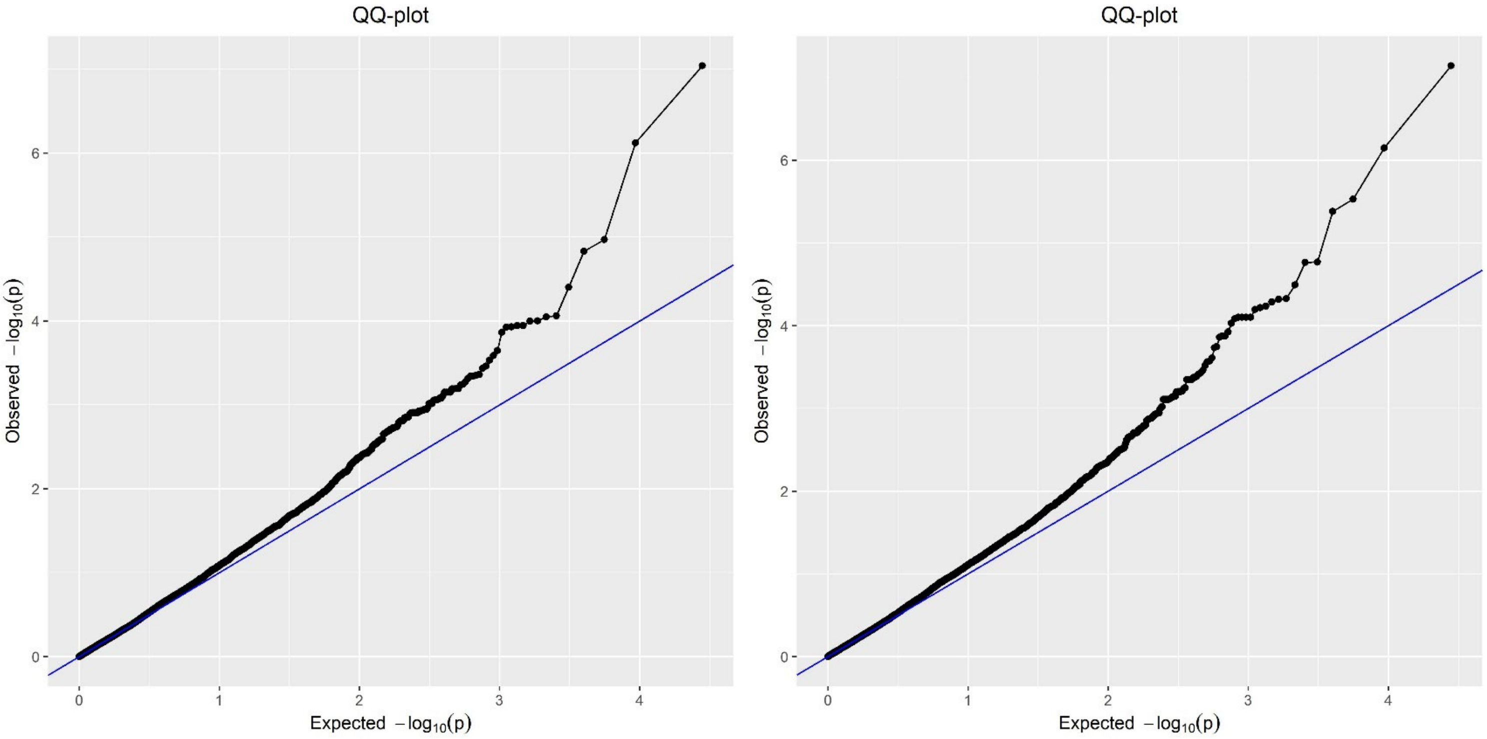

Supplement: Supplementary file 12 — Supplementary Figure S10 Comparison of Q-Q plots from 2017 GWAS (PDF 832 kb) [file 122_2022_4205_MOESM12_ESM.pdf]
