# Supplementary material for: Genetic dissection of morphological variation in rosette leaves and leafy heads in cabbage (Brassica oleracea var. capitata)
Source: Theor Appl Genet. 2022 Sep 3;135(10):3611–28. doi: 10.1007/s00122-022-04205-w (PMC9519658; doi:10.1007/s00122-022-04205-w)

Leaf Area (rosette stage). Population structure correction based on Principal Coordinates and Kinship (left), or Kinship only (right).

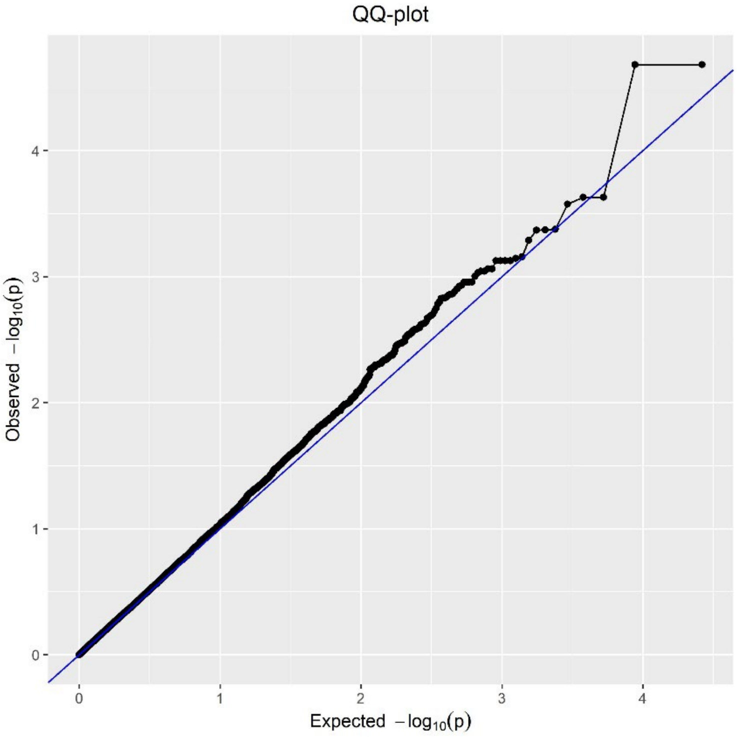

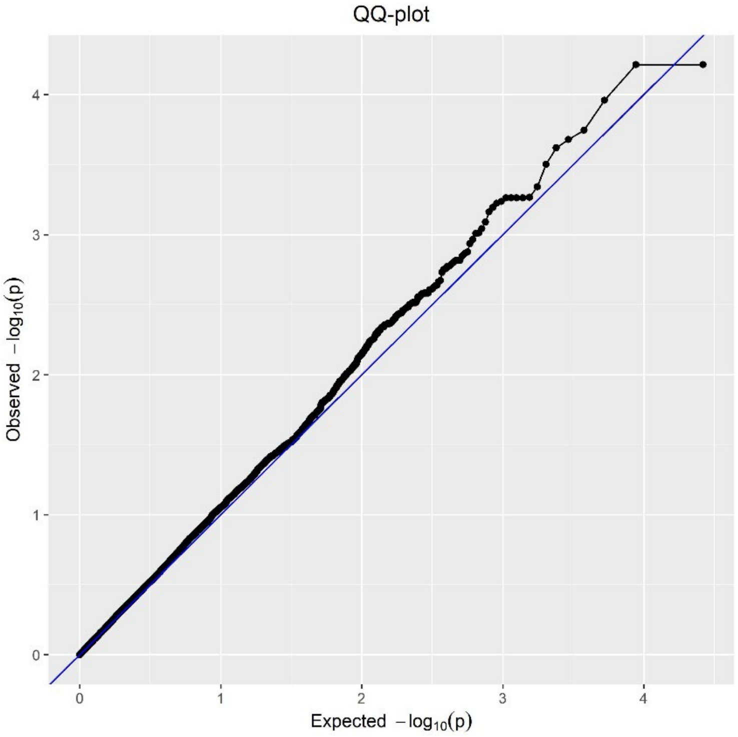

Leaf Width (rosette stage). Population structure correction based on Principal Coordinates and Kinship (left), or Kinship only (right).

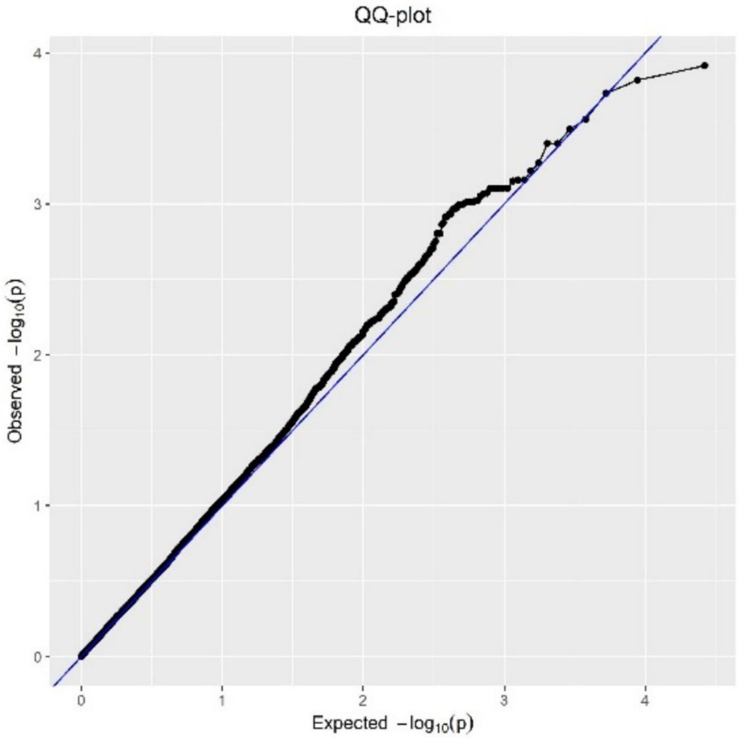

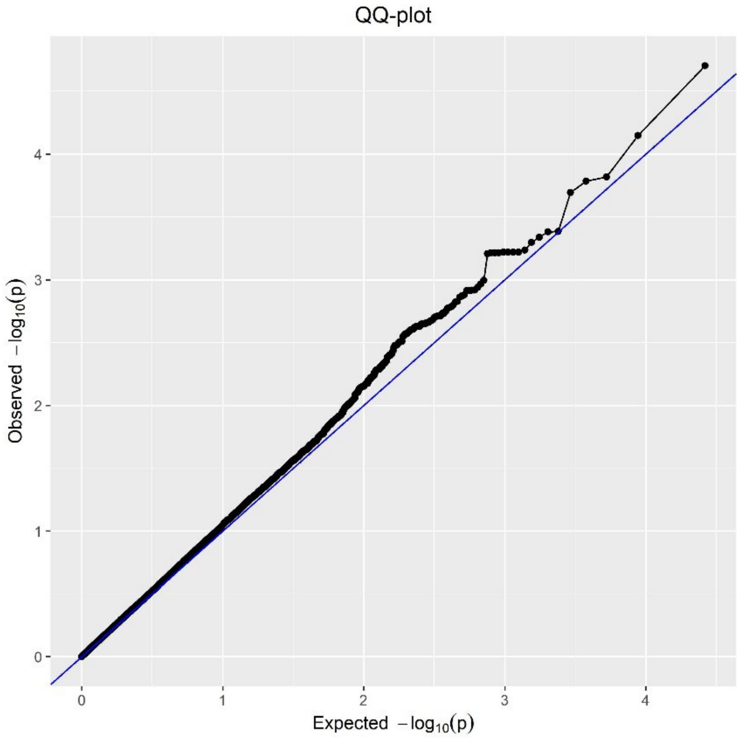

Leaf Length (rosette stage). Population structure correction based on Principal Coordinates and Kinship (left), or Kinship only (right).

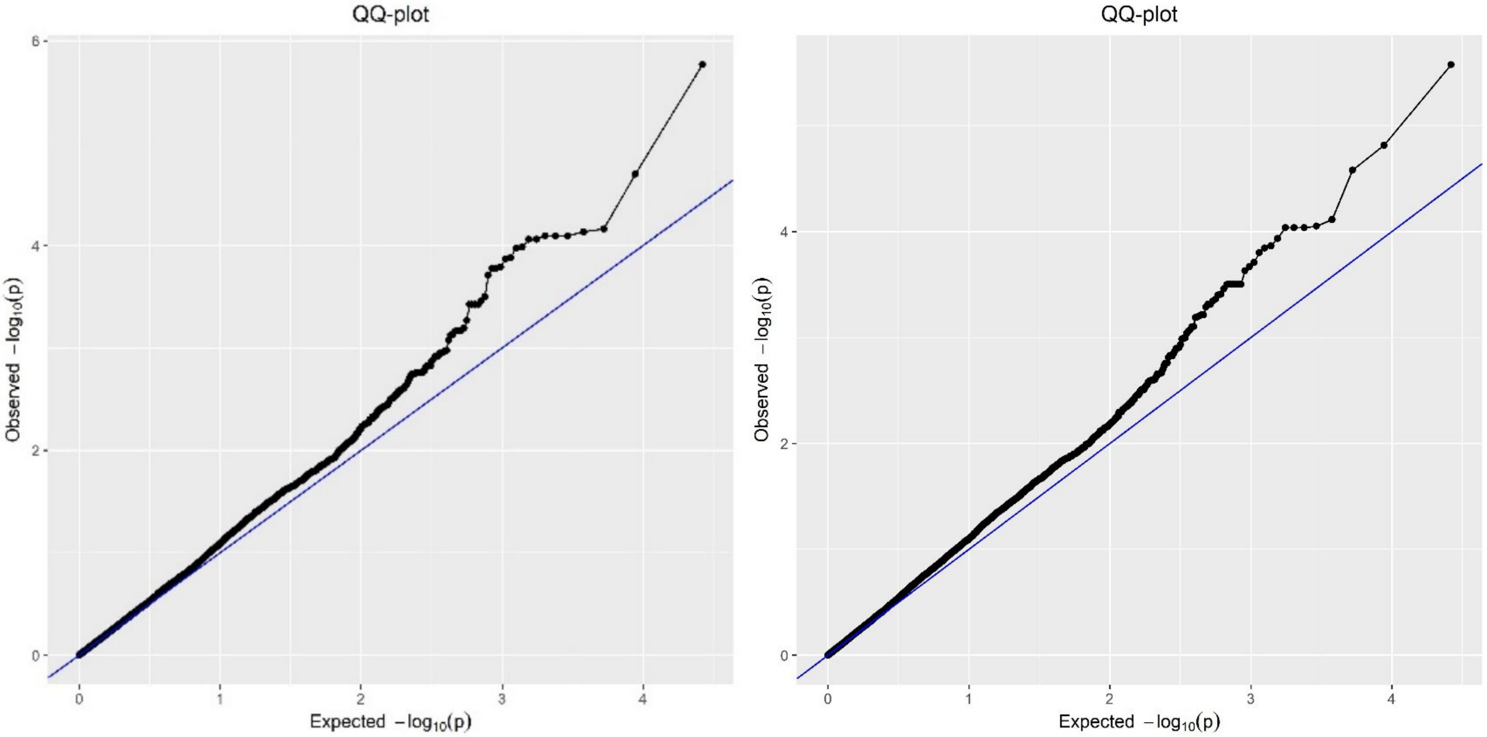

Leaf Ratio (rosette stage). Population structure correction based on Principal Coordinates and Kinship (left), or Kinship only (right).

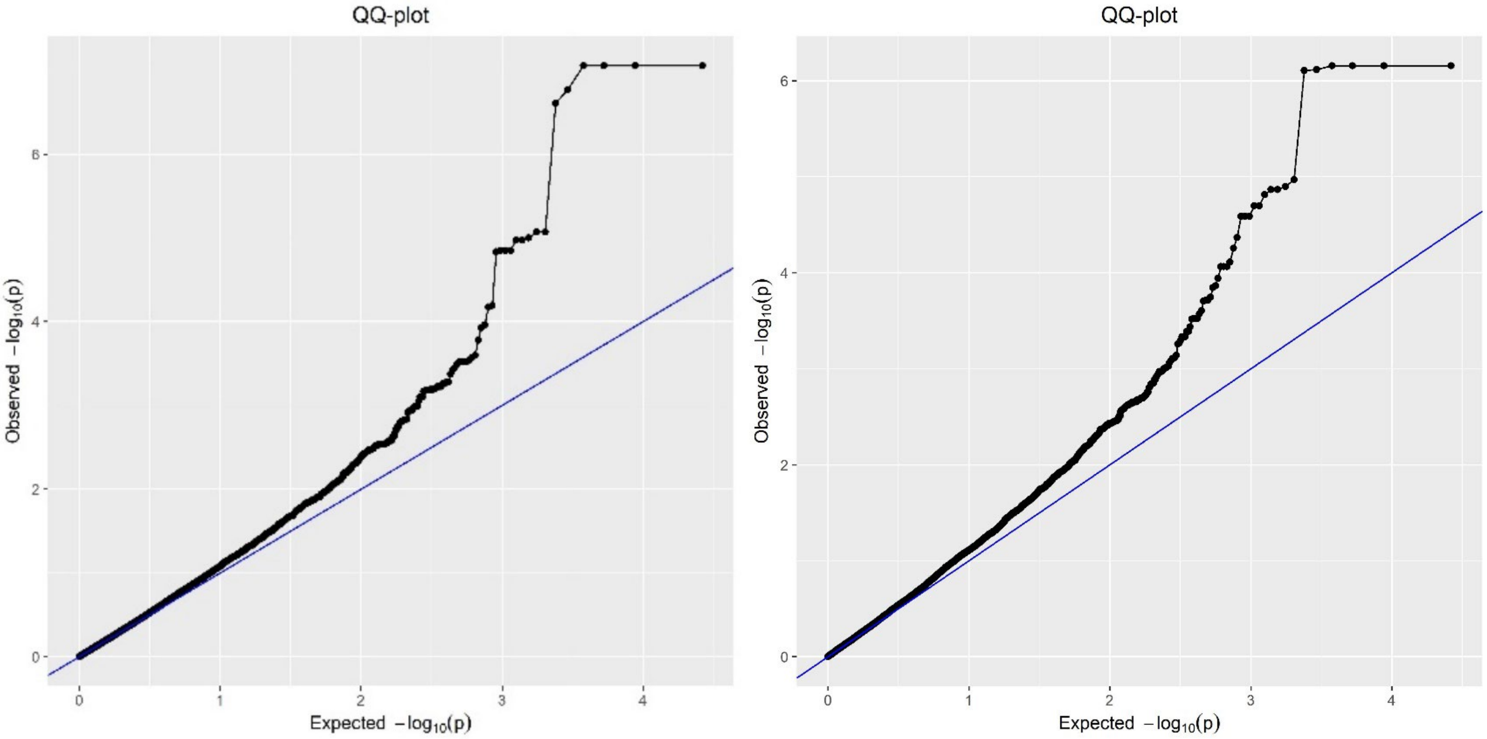

Leaf Area (heading stage). Population structure correction based on Principal Coordinates and Kinship (left), or Kinship only (right).

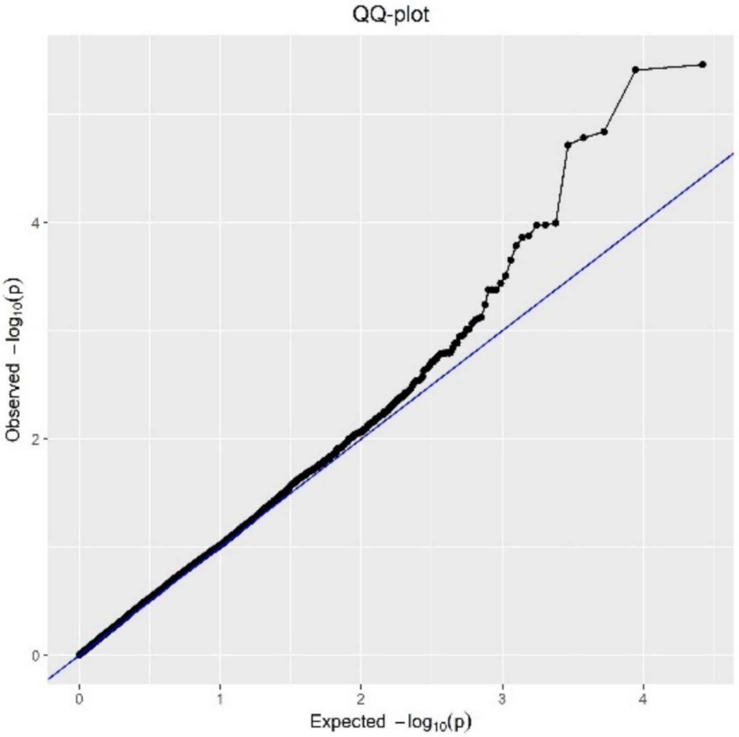

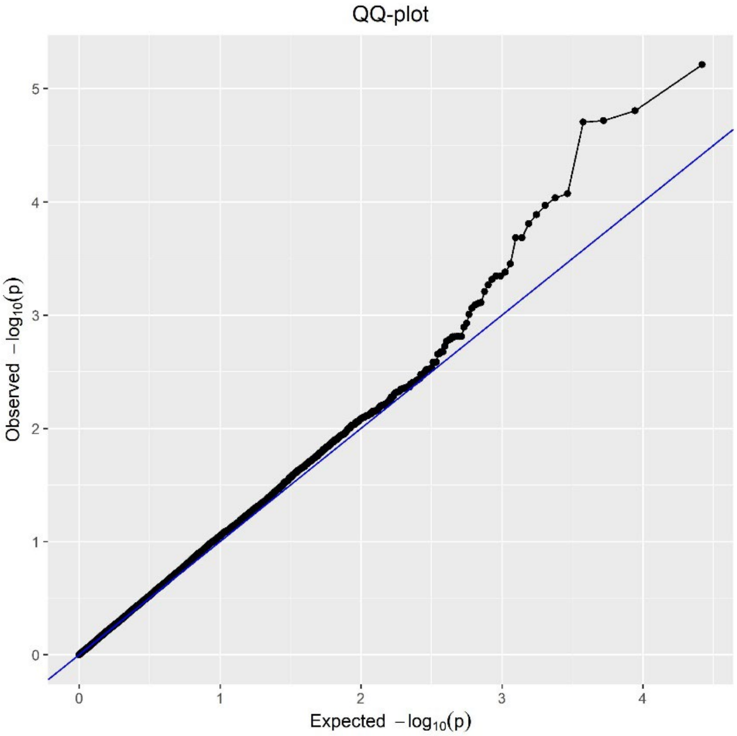

Leaf Width (heading stage). Population structure correction based on Principal Coordinates and Kinship (left), or Kinship only (right).

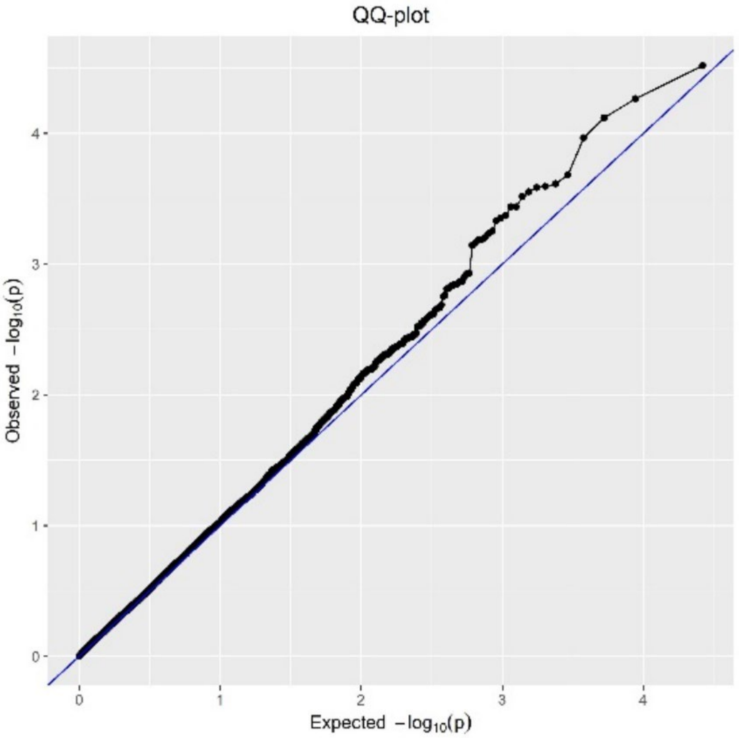

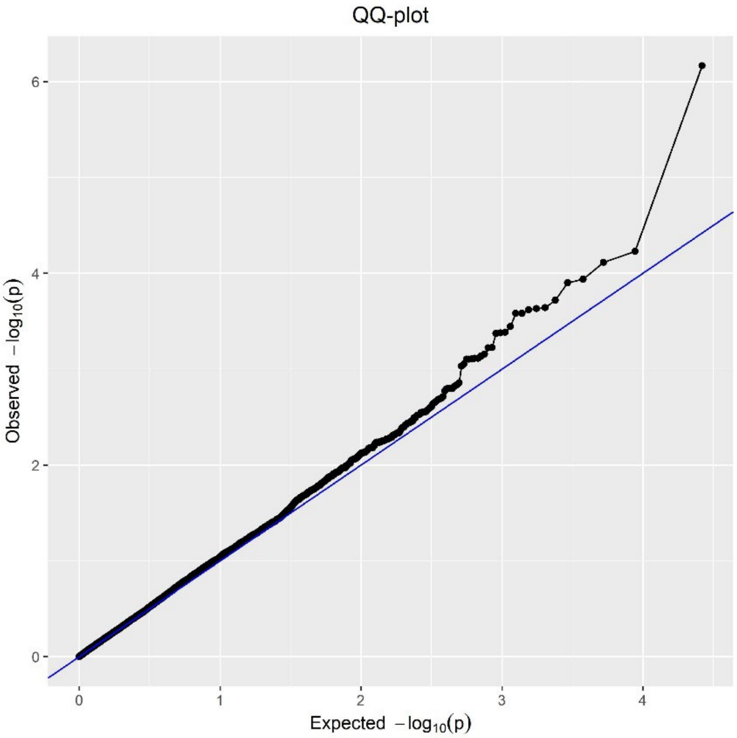

Leaf Length (heading stage). Population structure correction based on Principal Coordinates and Kinship (left), or Kinship only (right).

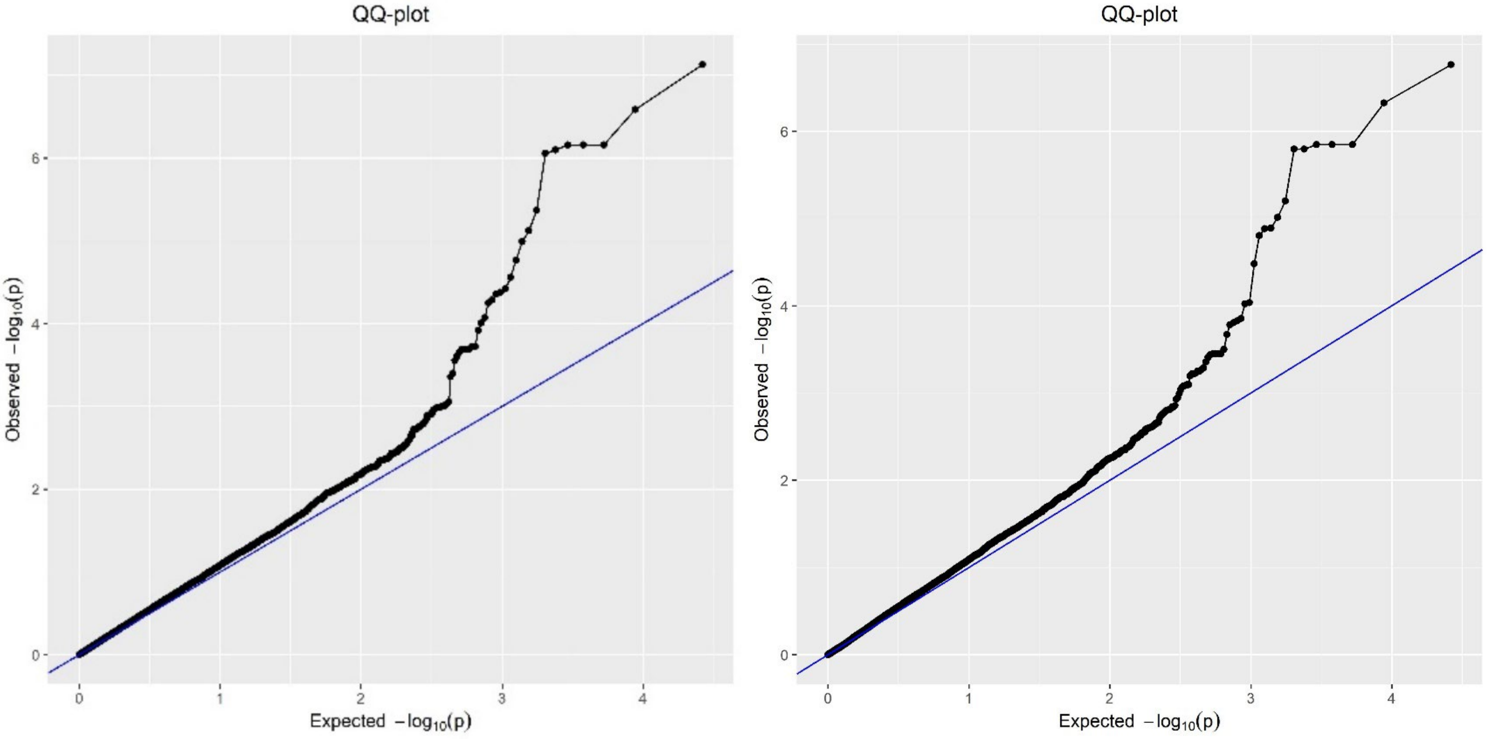

Leaf Ratio (heading stage). Population structure correction based on Principal Coordinates and Kinship (left), or Kinship only (right).

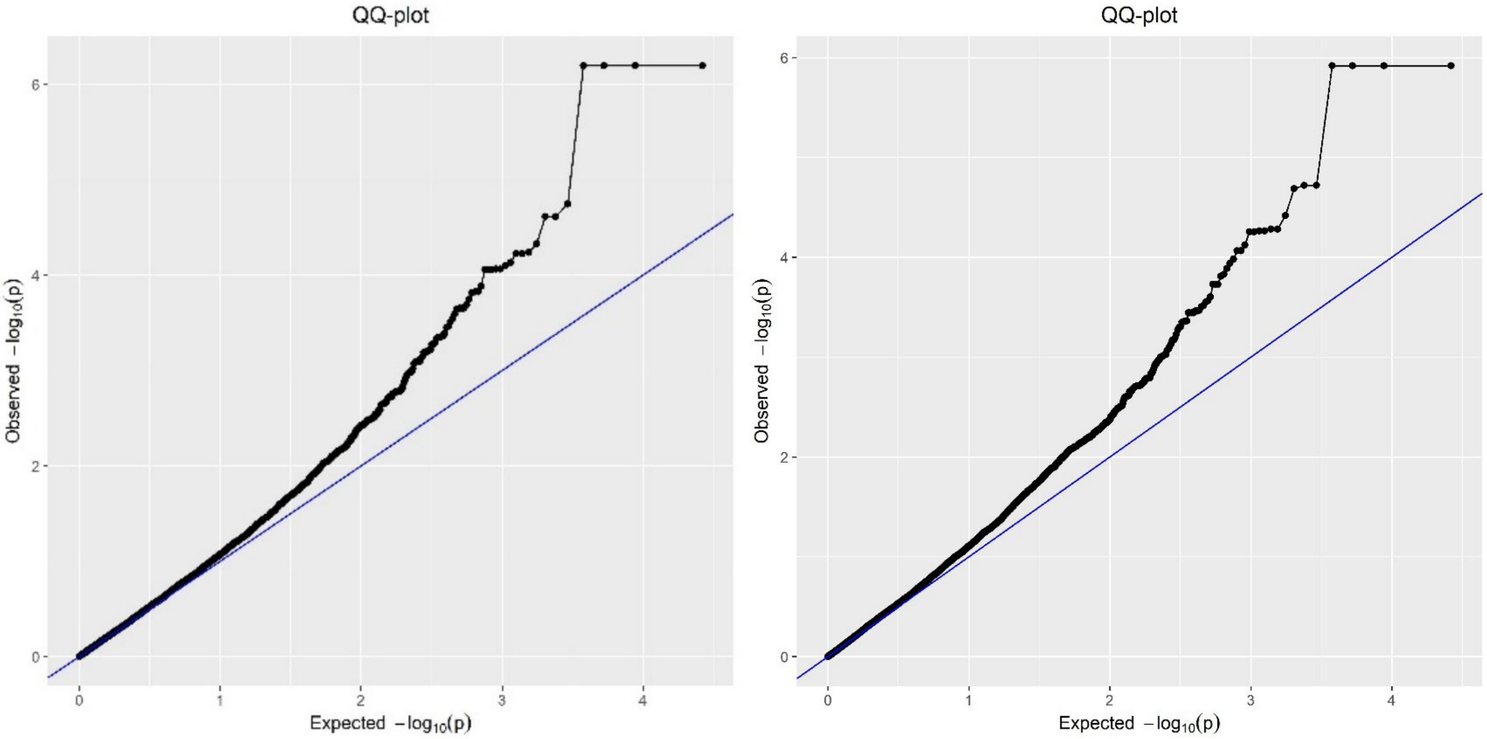

Rosette Leaf Number (heading stage). Population structure correction based on Principal Coordinates and Kinship (left), or Kinship only (right).

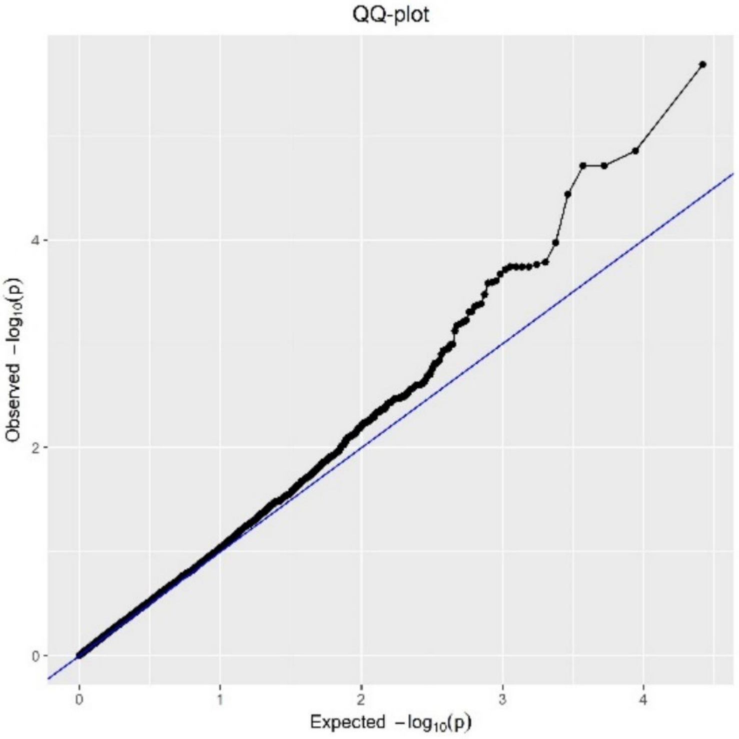

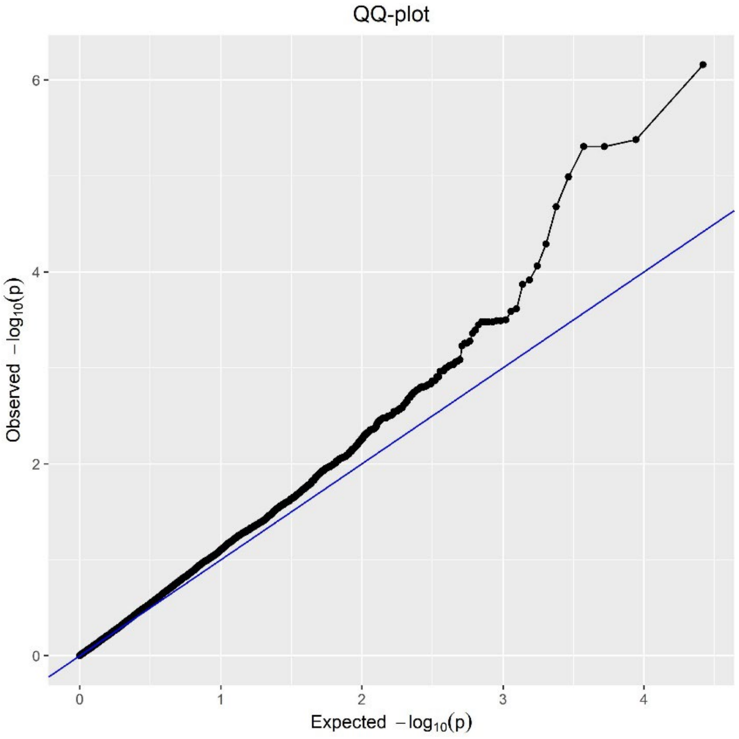

Head Area. Population structure correction based on Principal Coordinates and Kinship (left), or Kinship only (right).

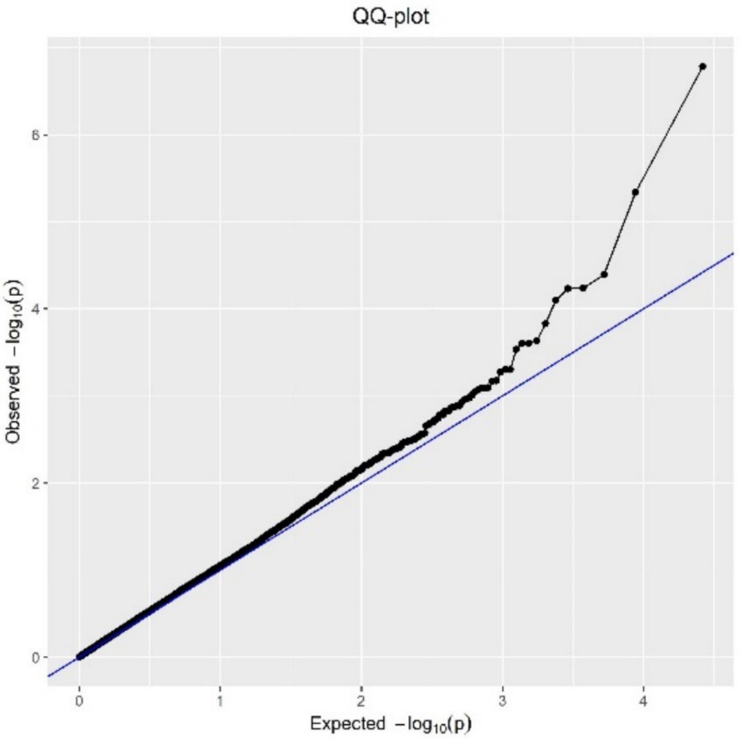

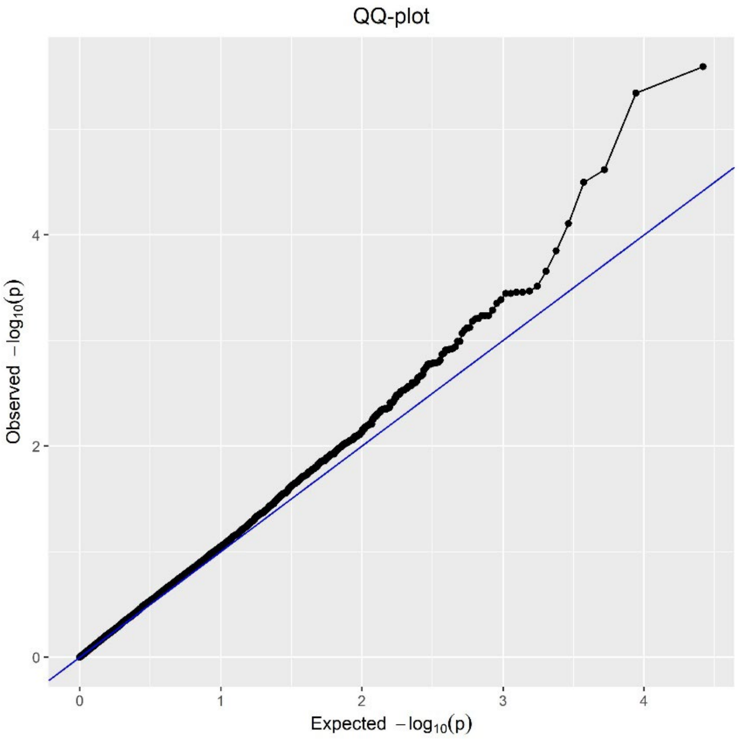

Head Width. Population structure correction based on Principal Coordinates and Kinship (left), or Kinship only (right).

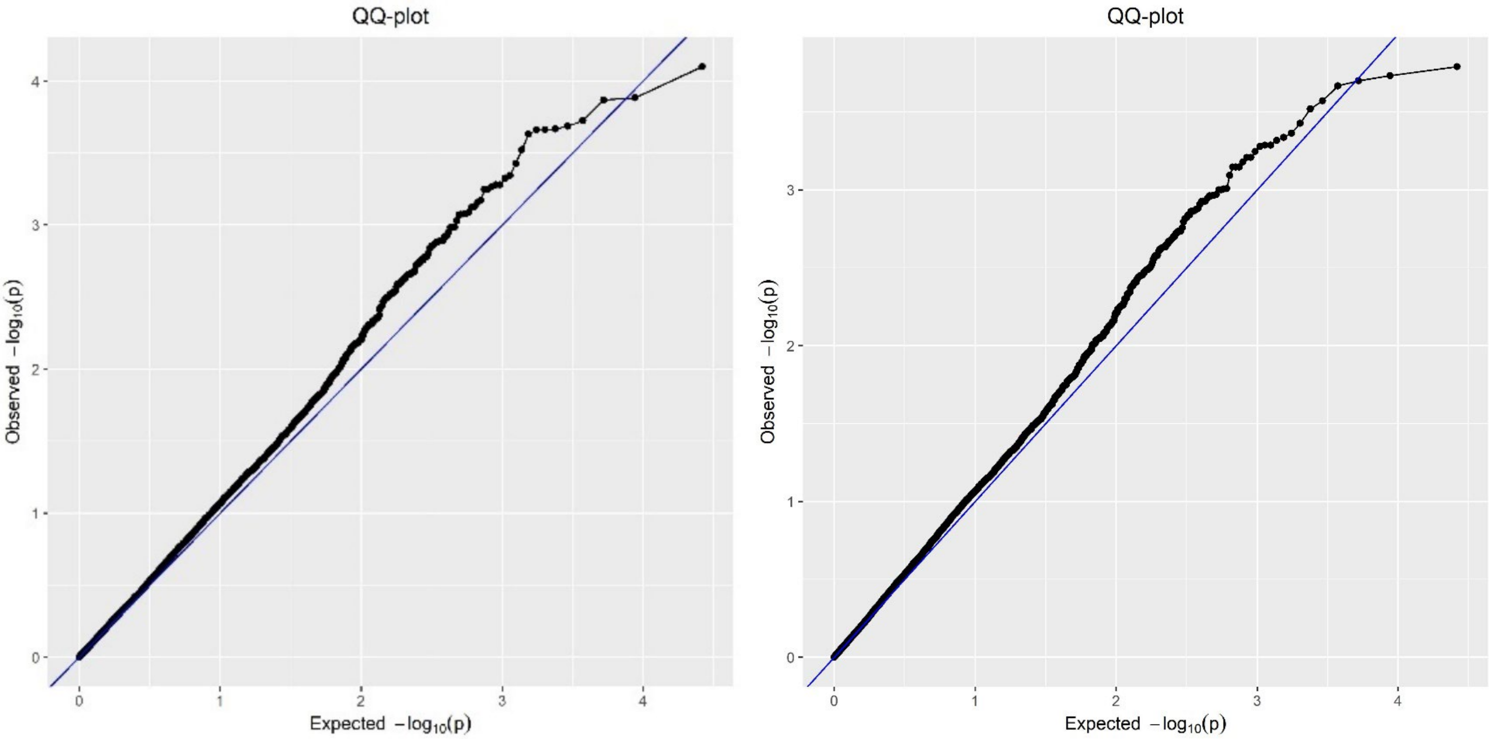

Head Height. Population structure correction based on Principal Coordinates and Kinship (left), or Kinship only (right).

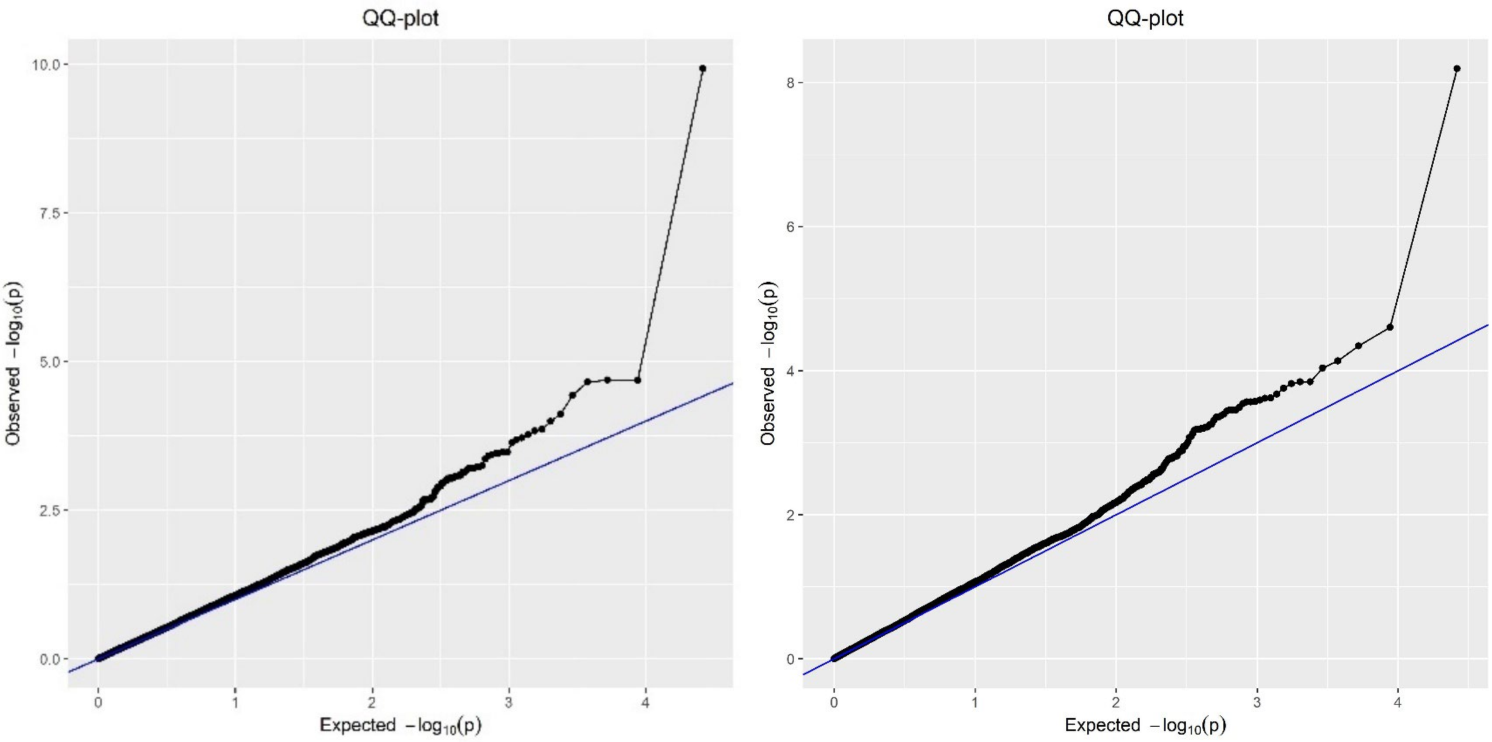

Head Ratio. Population structure correction based on Principal Coordinates and Kinship (left), or Kinship only (right).

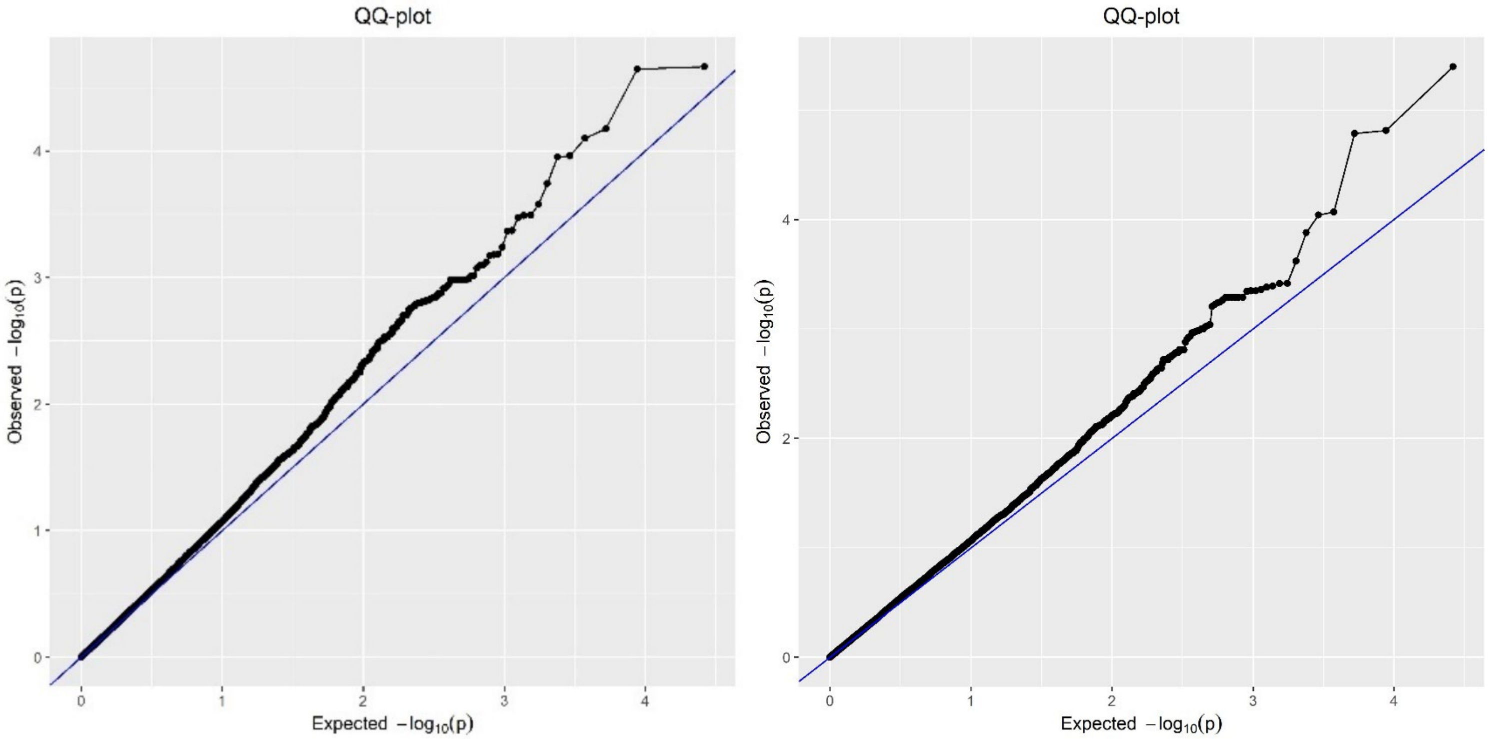

Head Weight. Population structure correction based on Principal Coordinates and Kinship (left), or Kinship only (right).

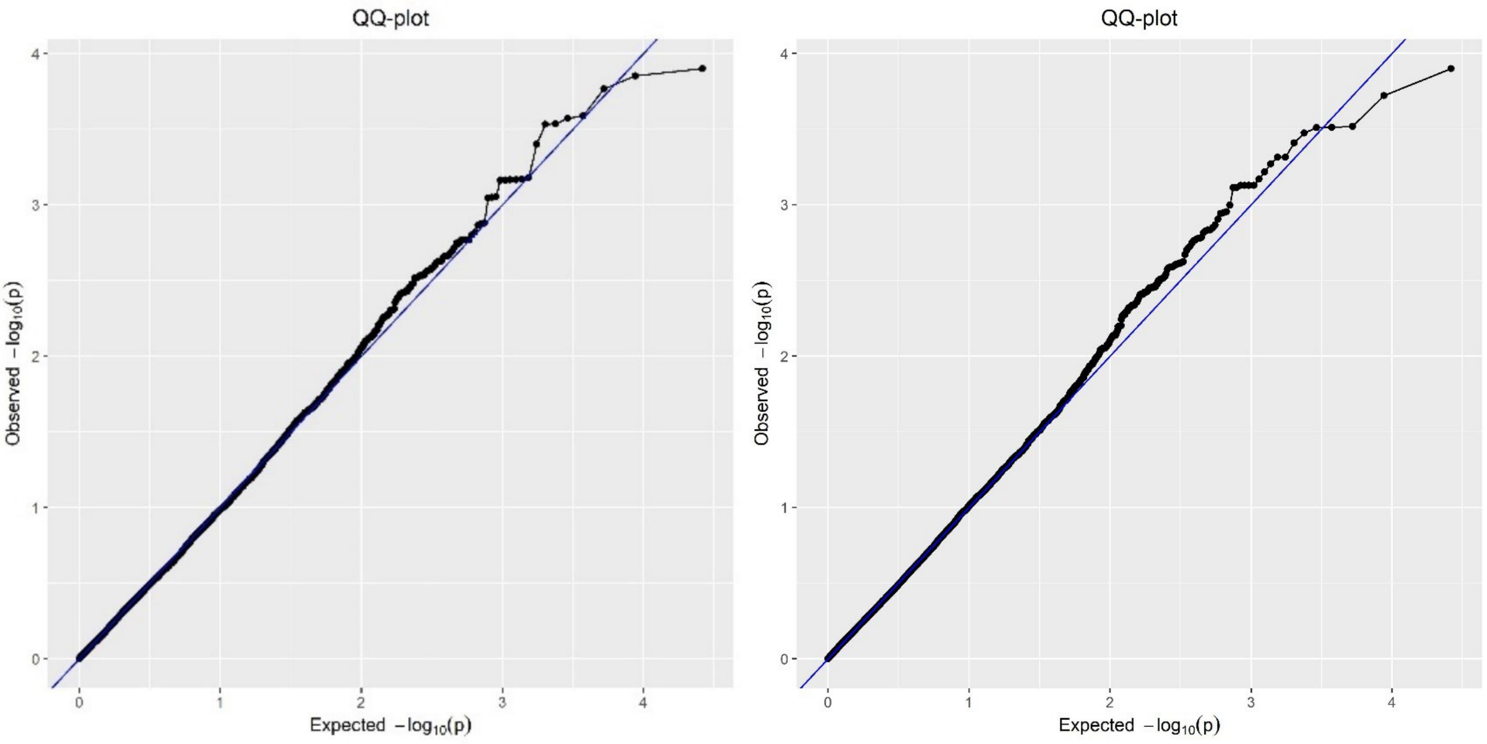

Supplement: Supplementary file 14 — Supplementary Figure S12 Comparison of Q-Q plots from 2019 GWAS (PDF 1304 kb) [file 122_2022_4205_MOESM14_ESM.pdf]
